# Supplementary material for: Quitting patient care and career break intentions among general practitioners in South West England: findings of a census survey of general practitioners
Source: BMJ Open. 2017 Apr 11;7(4):e015853. doi: 10.1136/bmjopen-2017-015853 (PMC5719652; doi:10.1136/bmjopen-2017-015853)
Supplement: Supplementary data [file bmjopen-2017-015853supp001.pdf]

Supplementary Online Material Table 1. Sample characteristics

Characteristic (N/3370)	Responded n (%)
**Gender (3370)**	
Male	1051 (67)
Female	1197 (66)
**Age (years; 3370)**	
Under 40	500 (54)
40-49	738 (68)
50-54	391 (72)
55-59	409 (76)
60-69	192 (74)
70 and over	18 (75)
**Employment Status (3370)**	
GP provider	1335 (71)
GP salaried	394 (57)
Non-principal/locum	519 (64)
**Survey sent by email (3370)**	
Yes	1304 (71)
No	944 (62)
**Postal address used (3370)**	
Practice address	1744 (67)
Home address	504 (64)

National Institute for Health Research | University of Exeter Medical School | University of Bristol | University of Exeter Business School | Centre for Workforce Intelligence

SUPPORTING THE GP WORKFORCE - ReGROUP QUESTIONNAIRE

This questionnaire is part of a research study examining the issue of the GP workforce. Please help by completing the questionnaire. Please answer the questions below by ticking one box for each question and printing text where required in block capitals. We will keep your answers completely confidential.

If you would prefer to complete the survey online, please go to ex.ac.uk/GP_Workforce

Reference 1234567890

'<u>Direct patient care</u>' includes time spent in all routine general practice activities (in the UK), including consultations, telephone, clinical and practice administration, prescribing and referral.

GP WORKFORCE QUESTIONS

Q.1 Please tell us which of the following scenarios applies to you?

☐ I am currently working in direct patient care
☐ I am currently on a career break (i.e. a break of 6 months or more for maternity/paternity leave or personal/professional development, including doing medical work abroad)
☐ I have permanently left direct patient care within the last 5 years.................................. **Go to Q.9**
☐ None of the above............................ **Go to Q.9**

Q.2 How likely is it that you will permanently leave direct patient care within the <u>next 2 years</u>?

☐ Very Likely
☐ Likely
☐ Unlikely
☐ Very unlikely

Q.3 How likely is it that you will permanently leave direct patient care within the <u>next 5 years</u>?

☐ Very Likely
☐ Likely
☐ Unlikely
☐ Very unlikely

Q.4 Overall, have you reduced your weekly average hours spent in direct patient care within the <u>last 5 years</u>?

☐ No
☐ Yes

Q.5 How likely is it that you will reduce your weekly average hours spent in direct patient care within the <u>next 5 years</u>?

☐ Very Likely
☐ Likely
☐ Unlikely

Q.6 How likely is it that you will take a career break (or another career break) within the <u>next 5 years</u>?

☐ Very Likely
☐ Likely
☐ Unlikely
☐ Very unlikely

Q.7 How would you describe your current level of morale?

☐ Very Low
☐ Low
☐ Neither low nor high
☐ High
☐ Very high

Q.8 Have you taken any steps toward changing your work-life balance (e.g. sought pension advice, consulted LMC, BMA etc)?

☐ No, I am still considering my options
☐ Yes, I am in the process of making this change
☐ Yes, I have made this change

ABOUT YOU

Q.9 What is your gender?

☐ Male
☐ Female
☐ Prefer not to say

Q.10 What is your age? (to nearest year)

[ ]

Q.11 What year did you qualify in medicine? (YYYY)

[ ]

Please turn over ☞

☐Very unlikely

Q.12 **What year did you qualify as a GP? (YYYY)**

Q.13 **From what region did you obtain your primary medical qualification?**

☐UK/Ireland
☐Europe (non-UK/Ireland)
☐South Asia
☐Other

Q.14 **What is your ethnic group?**

☐White
☐Mixed/multiple ethnic groups
☐Asian/Asian British
☐Black/African/Caribbean/Black British
☐Other ethnic group

Please write in:

Q.15 **In your current/most recent direct patient care role, what is/was your position?**

☐GP Partner
☐Salaried GP
☐Locum GP
☐Other

Please write in:

Q.16 **In your current/most recent direct patient role, how many sessions do/did you work in a typical week? (Please consider a session as a block of approximately four hours of clinical time)**

Please write in:

sessions

Q.17 **When do you work these sessions in a typical week? Please tick the appropriate boxes:**

	MON	TUE	WED	THU	FRI	SAT	SUN
Morning	☐	☐	☐	☐	☐	☐	☐
Afternoon	☐	☐	☐	☐	☐	☐	☐
Evening	☐	☐	☐	☐	☐	☐	☐

Q.18 **Do you have a practice that you are primarily affiliated to in your most recent direct patient care role?**

☐No
☐Yes

If yes, please write in the practice name AND ODS code (e.g. L123456), if known:

Q.19 **Are you personally involved in the delivery of out of hours direct patient care?**

☐Yes
☐No

CAN WE TALK TO YOU?

Q.20 **Would you be willing to take part in a confidential interview (45mins) to discuss issues around work-life balance? (You would be reimbursed for time)**

☐Yes
☐No

Q.21 **We plan on developing policies and strategies to help support the experienced GP workforce. Would you agree to be approached by this research team for other research studies that address this agenda?**

☐Yes
☐No

Q.22 **If you would be willing to be interviewed and/or be contacted for other research studies addressing the workforce agenda, please provide:**

Preferred method of contact

Your email address: ☐

____

Your telephone number: ☐

____

Q.23 **What is your preferred time of day to be contacted?**

☐Morning
☐Lunchtime
☐Afternoon
☐Evening

On:

☐Weekdays
☐Weekends

PRIZE DRAW

Q.24 **Please tick the box below if you would like to be entered into a prize draw to receive one of five Kindles**

☐Yes, please enter me into the prize draw

THANK YOU FOR YOUR TIME

Please return this questionnaire in the reply paid envelope provided (no stamp is needed)
