# Supplementary material for: Quitting patient care and career break intentions among general practitioners in South West England: findings of a census survey of general practitioners
Source: BMJ Open. 2017 Apr 11;7(4):e015853. doi: 10.1136/bmjopen-2017-015853 (PMC5719652; doi:10.1136/bmjopen-2017-015853)
Supplement: Author's manuscript [file bmjopen-2017-015853.draft_revisions.pdf]

BMJ Open

# Quitting patient care and career break intentions among general practitioners in South West England: findings of a census survey of GPs.



| | |
|---|---|
| Journal: | *BMJ Open* |
| Manuscript ID | bmjopen-2017-015853 |
| Article Type: | Research |
| Date Submitted by the Author: | 05-Jan-2017 |



| | |
|---|---|
| Complete List of Authors: | Fletcher, Emily; University of Exeter, Primary Care Research Group, University of Exeter Medical School<br>Abel, Gary; University of Exeter, Primary Care Research Group, University of Exeter Medical School<br>Anderson, Rob; University of Exeter, Evidence Synthesis & Modelling for Health Improvement, University of Exeter Medical School<br>Richards, Suzanne; University of Exeter Medical School, Primary Care Research Group, University of Exeter Medical School<br>Salisbury, Chris; University of Bristol, Centre for Academic Primary Care, School of Social and Community Medicine<br>Dean, Sarah; University of Exeter, Psychology Applied to Rehabilitation and Health, University of Exeter Medical School<br>Sansom, Anna; University of Exeter Medical School, Primary Care<br>Warren, Fiona; University of Exeter Medical School, Institute of Health Research<br>Campbell, John; University of Exeter, Primary Care Research Group, University of Exeter Medical School |



| | |
|---|---|
| <b>Primary Subject Heading</b>: | General practice / Family practice |
| Secondary Subject Heading: | Health services research |
| Keywords: | PRIMARY CARE, GENERAL PRACTICE, WORKFORCE, MORALE, SURVEY, Organisation of health services < HEALTH SERVICES ADMINISTRATION & MANAGEMENT |
| | |


SCHOLARONE™
Manuscripts

**Quitting patient care and career break intentions among general practitioners in South West England: findings of a census survey of GPs.**

Emily Fletcher *research fellow*[1], Gary Abel *statistician*[1], Rob Anderson *associate professor*[2] Suzanne H Richards *senior lecturer*[1], Chris Salisbury *professor of primary health care*[3], Sarah Dean *associate professor*[4], Anna Sansom *research fellow*[1], Fiona Warren *statistician*[1], John L Campbell *professor of general practice and primary care*[1*]

[1] Primary Care Research Group, University of Exeter Medical School, Exeter, UK

[2] Evidence Synthesis & Modelling for Health Improvement, University of Exeter Medical School, Exeter, UK

[3]Centre for Academic Primary Care, NIHR School for Primary Care Research, School of Social and Community Medicine, University of Bristol, Bristol, UK

[4]Psychology Applied to Rehabilitation and Health, University of Exeter Medical School, Exeter, UK

*Corresponding author:

Professor John Campbell
Primary Care Research Group
University of Exeter Medical School
Smeall Building, St Luke's Campus
Magdalen Road
Exeter
EX1 2LU
john.campbell@exeter.ac.uk

Word count (excludes title page, abstract, references, figures, tables):

Key words: primary care; general practice; workforce; morale; survey; organisation of care



For peer review only

For peer review only - http://bmjopen.bmj.com/site/about/guidelines.xhtml

## ABSTRACT

**Objective** Given recent concerns regarding general practice (GP) workforce capacity, we aimed to describe GPs' career intentions, especially those which might impact on GP workforce availability over the next 5 years.

**Design** Census survey, conducted between April and May 2016 using postal and online responses, of all GPs on the NHS Performers list and eligible to practise in primary care. Two reminders were used as necessary.

**Setting** South West England (population 3.5 million), a region with low overall socio-economic deprivation.

**Participants** 2248 out of 3370 eligible GPs (67% response rate).

**Main outcome measures** Reported likelihood of permanently leaving or reducing hours spent in direct patient care or of taking a career break within the next five years, and present morale weighted for non-response.

**Results** Responders included 2179 GPs engaged in patient care. Of these, 863 (37% weighted, 95%CI 35%, 39%) reported a high likelihood of quitting direct patient care within the next five years. Overall, 1535 (70% weighted, 95%CI 68%, 72%) respondents reported a career intention that would negatively impact GP workforce capacity over the next five years, through permanently leaving or reducing hours spent in direct patient care, or through taking a career break. GP age was an important predictor of career intentions; sharp increases in the proportion of GPs intending to quit patient care were evident from 52 years. Only 305 (14% weighted, 95%CI 13%, 16%) of GPs' reported high morale, while 54% (weighted, 95%CI 52%, 56%) reported low morale. Low morale was particularly common amongst GP partners. Current morale strongly predicted GPs' career intentions; those with very low morale were particularly likely to report intentions to quit patient care or to take a career break.

**Conclusions** A substantial majority of GPs in South West England report low morale. Many are considering career intentions which, if implemented, would adversely impact GP workforce capacity within a short time period.



For peer review only - http://bmjopen.bmj.com/site/about/guidelines.xhtml

**Study registration** NIHR HS&DR - 14/196/02, UKCRN ID 20700

**ARTICLE SUMMARY**

Strengths and limitations of this study

- Census survey of all GPs in South West England, providing a cross-sectional overview of quit and career break intentions.
- High response rate, reflecting both rigorous planning and implementation of the survey, and interest among GPs in the subject matter of workforce challenges.
- The potential for non-response bias was reduced by weighting estimates to account for differential response rates by age, gender and practice role.
- The survey was cross-sectional, rather than longitudinal in design; previous work demonstrates that intention to quit is a strong predictor of actually leaving.
- The research was undertaken within a rapidly changing GP workforce policy and practice environment.

For peer review only



For peer review only - http://bmjopen.bmj.com/site/about/guidelines.xhtml

**INTRODUCTION**

UK healthcare has traditionally had a strong primary care base in which general practitioners (GPs) have a central role in providing care across many settings, including within general practices, in out-of-hours care, and in walk-in centres. Ninety percent of UK NHS patient contact takes place within primary care – 1.3m consultations every working day, 340m consultations per year, and with a projected primary care workload of 430m consultations per year by 2018(1, 2). Around 74% of primary care contacts take place with a general practitioner (GP). General practice has been described as "the jewel in the crown" of the NHS(3). GPs are trained, and have particular abilities in the diagnosis and management of patients with complex multi-morbidity.

UK general practice is, however, facing major problems regarding maintaining the GP workforce, with imminent GP shortages and a concomitant potential risk to patient care. A near quadrupling of unfilled GP posts was observed between 2010 and 2013 (from 2.1% to 7.9%), associated with an overall reduction in the number of GPs in England from 62 per 100,000 in 2009 to 59.5 in 2013(4). An estimated 12% of 2,947 GP training places were unfilled in England in 2013/14. These issues are compounded by an ageing GP workforce (30% of the 43,000 current GPs are over 50 years old(4)). Workforce issues are especially pertinent in inner city settings where recruitment and retention difficulties are further exacerbated by issues relating to the socio-demographic mix of the population and to increased demands for care.

Research commissioned by the BMA has highlighted the continuing recruitment and retention challenges. In a 2014 study of 431 doctors from the 2006 cohort of medical graduates, those in general practice reported the lowest morale, with higher than expected workload being identified as a key problem(5). Of male doctors, 90% plan to work full time compared with just 40% of female doctors(4). Younger GPs currently appear to be more reluctant to take on the financial risks and responsibilities of becoming a partner in a practice, leading to existing partners experiencing an increased workload(6). Recent surveys and reports have suggested that many GPs are considering permanently leaving direct patient care (1, 4, 7). Published evidence suggests that nearly half of GPs leaving general practice between 2009 and 2014 were under 50 years old(8).

There is thus an urgent need to find ways of retaining the GP workforce. If unaddressed, "meltdown" in NHS care may follow within the foreseeable future(1). The situation has been described as a "crisis"(9, 10) and there has been a call for policies and strategies to help retain GPs in direct patient care(4, 11). The future of NHS care over the next five years(12) is likely to involve new models of care, with innovations involving increasing professional skill mix and new approaches to managing



For peer review only - http://bmjopen.bmj.com/site/about/guidelines.xhtml

the service(12, 13) and in establishing federations of previously independent practices(12). It is vital that the GP workforce is sustained through this period of change.

Our research targets the critically important area of retention of experienced GPs in direct patient care, especially focussing on those GPs who may be considering retirement, and those GPs who have taken a career break (most often on account of family reasons). Following piloting of our questionnaire in 2015(14), this paper reports the findings of a recent census survey in which we aimed to describe GPs' career intentions, especially those which might impact on GP workforce availability over the next five years. The survey constitutes one part of a comprehensive programme of work, seeking to identify implementable policies and strategies to support the retention of experienced GPs in direct patient care and to support the return of GPs following a career break.

## METHODS

### Study sample

The sampling frame was the Medical Performers List of all GPs registered to practise in South West England, an area with a population of 3.5 million patients. National Performers Lists provide reassurance for the public that GPs, Dentists and Opticians practising in the NHS are suitably qualified, have up to date training, regulatory checks and appropriate English language skills(15). There were no exclusion criteria. The survey was administered between April and June 2016.

### Questionnaire

The questionnaire was based upon that used within earlier work (14), modified to increase the number of questions from 11 to 24, including rewording of four questions to reflect alignment of wording with other questionnaires of broadly similar intent, and by providing clear definitions for key concepts in the questionnaire including a career break, taking steps toward changing work-life balance and defining a clinical session. The questionnaire (Supplementary Online Material) comprised items that asked GPs about their career intentions, reporting on the likelihood that they would permanently leave direct patient care within the next two years, or within the next five years. GPs were also asked to report the likelihood that they would take a career break within the next five years, or that they would reduce their weekly average hours spent in direct patient care during this time period. GPs rated the likelihood of these events from "very likely" to "very unlikely" using a 4-point scale. The questionnaire also included a question about current level of morale, and captured general demographic data: gender, age, ethnicity, region and year of graduation, and current GP employment status (e.g., partner, salaried), number and pattern of sessions worked in a typical week, and involvement in delivering out-of-hours care.



For peer review only - http://bmjopen.bmj.com/site/about/guidelines.xhtml

**Data collection**

GPs were sent study materials through the post either to their practice or home address, and also by email, where available. The questionnaire was available for completion by post and online. The survey was supported by a comprehensive strategy of publicising the research through routine newsletters and circulars of relevant organisations and networks, including Local Medical Committees, Clinical Research Networks, Health Education England South West, the Royal College of General Practitioners, University of Exeter Medical School, and the South West Academic Health Science Network.

If a GP returned multiple online or postal surveys only the first response received by the research team was analysed. Postal response data were double entered and discrepancy checking was undertaken. Response data were stored securely and without participant name or address.

**Patient involvement**

Although the study participants were GPs rather than patients, patient representatives contributed to the design of the survey. The planned work was presented to the wider project's Patient and Public Involvement (PPI) group(16), by way of sharing the process and to check the integrity of the work, and the group provided supportive feedback. The survey results were presented at a project management group meeting, which included PPI representatives who directly contributed to interpreting and contextualising the results.

**Statistical analysis**

Differential response rates between different groups of GPs would potentially introduce bias into crude survey findings. To counter this we employed non-response weights. Inverse probability weights were calculated based on three factors: age (<40, 40-49, 50-54, 55-59, and 60 years and over), gender (male and female), and role (partner, salaried, and locum/other). By employing these weights we estimated what responses we would have received with a 100% response rate under the assumption that non-responders would have responded similarly to GPs of the same age, gender and role. Logistic regression was used to investigate the association between responses to questions regarding future career intentions (permanently leaving direct patient care within the next two and five years, taking a career break within the next five years, and reducing average hours spent in direct patient care within the next five years). Each of the four sets of responses was dichotomised into 'very likely' and 'likely' versus other responses. Initially, unadjusted associations were examined for effects attributable to the explanatory factors of gender, age, country of qualification, ethnicity, role/position, and rating of current morale. Subsequently, regression models adjusting simultaneously for all explanatory factors were used to examine adjusted associations. Similar



For peer review only - http://bmjopen.bmj.com/site/about/guidelines.xhtml

models were used with reported morale as the outcome (but not including morale as an explanatory factor). Regression analyses were restricted to those respondents with complete data on gender, age, country of qualification, ethnicity, role/position and rating of current morale.

**Supplementary analysis**

Interactions were explored between various factors in the models. Whilst some of these were found to be statistically significant, the magnitude of the interaction terms was generally small and did not alter the interpretation of the data, with one exception commented on in the results. For this reason we have not reported the more complex interaction models here.

In addition, we considered the possibility that some groups of GPs, for example female GPs, may not report the intention to reduce hours spent in direct patient care because they already work fewer hours, on average, than other groups. We therefore performed two supplementary analysis, including either a binary variable indicating part-time working (defined as working less than 8 sessions per week) or a continuous variable detailing the reported number of sessions worked per week.

All analyses were performed in Stata 14.2.

**RESULTS**

Questionnaires were distributed by post to 3370 GPs, with 1841 (55%) GPs also sent the questionnaire by email (Supplementary Online Material Table 1). Completed questionnaires were received from 2248/3370 GPs who were surveyed (response rate 67%). Of the 2248 GP respondents, 673 (30%) used the online survey. Response rates were as high for both males and females (67% and 66% respectively). Participation was lower amongst GPs aged under 40 years (54%) when compared with the older GPs aged 50 and over (in excess of 68% in each age group), and was lower for salaried GPs (57%) when compared with GP partners (71%) and non-principal/locum GPs (64%).

The median age of respondents was 48 years (Table 1; interquartile range (IQR) 40 to 55, range 28 to 84 years). Eighty-five percent of respondents reported having a practice with which they were primarily affiliated; 25% of respondents reported that they were involved in the delivery of out-of-hours primary medical care. The majority (62%) of respondents were partners in their practices.



For peer review only - http://bmjopen.bmj.com/site/about/guidelines.xhtml

**Table 1 Characteristics of responding GPs (N=2248)**

| | | Proportion; n (%) |
|---|---|---|
| Gender | Male | 1053 (46.8) |
| | Female | 1190 (52.9) |
| | Prefer not to say | 3 (0.1) |
| | Missing | 2 (0.1) |
| Age (years) | Under 40 | 497 (22.1) |
| | 40-49 | 735 (32.7) |
| | 50-54 | 394 (17.5) |
| | 55–59 | 408 (18.2) |
| | 60 or over | 209 (9.3) |
| | Missing | 4 (0.2) |
| | Spoiled | 1 (0.0) |
| Ethnic group | White | 2100 (93.4) |
| | Mixed/multiple ethnic groups | 29 (1.3) |
| | Asian/Asian British | 78 (3.5) |
| | Black/African/Caribbean/Black British | 9 (0.4) |
| | Other ethnic group | 19 (0.9) |
| | Missing | 13 (0.6) |
| Region of qualification | UK/Ireland | 2107 (93.7) |
| | Europe (non-UK/Ireland) | 70 (3.1) |
| | South Asia | 21 (0.9) |
| | Other | 42 (1.9) |
| | Missing | 7 (0.3) |
| | Spoiled | 1 (0.0) |
| Role/position | GP Partner | 1403 (62.4) |
| | Salaried GP | 454 (20.2) |
| | Locum GP | 287 (12.8) |
| | Other | 68 (3.0) |
| | Missing | 7 (0.3) |
| | Spoiled | 29 (1.3) |

**Career intentions**

Of the 2248 responding GPs, 55 had already permanently left direct patient care (Table 2); 16 had selected 'none of the above' to reflect their working status in direct patient care – i.e. that they were not currently working in direct patient care, nor on a career break, but had not permanently quit – and were removed from further analysis. Differential response by age and role meant that GP groups reporting an intention to quit direct patient care were somewhat overrepresented, introducing a small bias into the crude results which is accounted for in the weighted percentages.

Of the 2179 GP participants included in the analysis, 473 (weighted percentage 20.3%, 95%CI 18.7%, 22.0%) reported a high likelihood of quitting direct patient care ('likely' or 'very likely') within the next two years, and 863 (weighted percentage 36.7%, 95%CI 34.7%, 38.8%) within the next five years (Table 2). There were 1252 (weighted percentage 56.7%, 95%CI 54.5%, 58.8%) participants who reported being likely or very likely to reduce hours, and 770 (weighted percentage 36.2%, 95%CI 61.6%, 65.8%) who reported the intention to take a career break within the next five years.



For peer review only - http://bmjopen.bmj.com/site/about/guidelines.xhtml

Considered together, 1535 (weighted percentage 69.9%, 95%CI 67.9%, 71.8%) participants reported that they were likely/very likely to pursue a career intention (one or more of the four presented) that would potentially adversely impact the workforce available in South West England within the next five years. The majority of participants also had low morale, with a substantially greater proportion of participants (1195 - weighted percentage 54.4%, 95%CI 52.2%, 56.5%) reporting 'low' or 'very low' levels of morale compared with only 305 (weighted percentage 14.2%, 95%CI 12.7%, 15.7%) reporting 'high' or 'very high' morale.

**Associations between GP characteristics and career intentions/morale**

Figure 1 illustrates the reported intention to quit direct patient care of responding GPs broken down by gender and each year of GP age, aggregating likely and very likely responses together. Reported quit intentions are strongly related to age, remaining low (<20%) among younger GPs (45 years and under) for quitting direct patient care in both of the next two and five years. Both of these outcomes show a sharp rise from the age of 52 years, with the proportion stating they were likely to quit in five years rising to almost 90% by the age of 56 years. The proportion anticipating taking a career break as 'likely' was highest for the youngest GPs, especially amongst younger women where nearly 9 out of 10 female GPs aged 30 years reported the intention to take a career break, presumably on account of anticipated maternity leave.

Of the 2179 respondents reporting that their current role involved direct patient care, 2119 (97%) provided complete information on gender, age, region of qualification, ethnicity, role/position and rating of current morale and were included in the regression analyses. Tables 3 and 4 show the results of the unadjusted and adjusted logistic regression analyses respectively, for career intentions. In unadjusted analyses there is strong evidence (p<0.001 for all) that gender and role are associated with being likely to report the intention to leave patient care within both two and five years, with female GPs being less likely to quit patient care than male GPs (OR 0.55 95% CI 0.46, 0.66 for leaving patient care in five years), and with locum GPs being the most likely to quit patient care and salaried GPs least likely. However, these associations are likely be confounded, for example by the fact younger doctors were more likely to be female compared with older doctors. Once adjustment is made for all other factors, the effects of these characteristics on intentions to quit are no longer significant. However, after adjustment for other variables, women were still substantially less likely to report intending to reduce hours spent in direct patient care (OR 0.58 95% CI 0.47, 0.71), and locum GPs remained more likely to report intending to take a career break when compared with GPs in other roles.



For peer review only - http://bmjopen.bmj.com/site/about/guidelines.xhtml

**Table 2 Career intentions of responding GPs (n=2179)**

| | How likely is it that you will permanently leave direct patient care within the next 2 years? | | | How likely is it that you will permanently leave direct patient care within the next 5 years? | | | How likely is it that you will reduce your weekly average hours spent in direct patient care within the next 5 years? | | | How likely is it that you will take a career break (or another career break) within the next 5 years? | | |
|---|---|---|---|---|---|---|---|---|---|---|---|---|
| | n | % crude | % Weighted (95% CI) | n | % crude | % weighted (95% CI) | n | % crude | % weighted (95% CI) | n | % crude | % weighted (95% CI) |
| Very likely | 255 | 11.7 | 10.8 (9.6, 12.1) | 607 | 27.9 | 25.4 (23.6, 27.2) | 773 | 35.5 | 34.0 (32.0, 36.0) | 453 | 20.8 | 20.7 (19.0, 22.5) |
| Likely | 218 | 10.0 | 9.5 (8.4, 10.8) | 256 | 11.7 | 11.4 (10.1, 12.8) | 479 | 22.0 | 22.7 (20.9, 24.5) | 317 | 14.5 | 15.6 (14.0, 17.2) |
| Unlikely | 795 | 36.5 | 36.6 (34.6, 38.7) | 675 | 31.0 | 32.3 (30.3, 34.3) | 585 | 26.8 | 27.5 (25.7, 29.5) | 756 | 34.7 | 34.9 (32.9, 36.9) |
| Very unlikely | 899 | 41.3 | 42.6 (40.5, 44.7) | 622 | 28.5 | 30.1 (28.2, 32.1) | 326 | 15.0 | 15.2 (13.7, 16.8) | 614 | 28.2 | 27.3 (25.4, 29.2) |
| Missing | 12 | 0.6 | 0.5 (0.3, 1.0) | 17 | 0.8 | 0.8 (0.0, 0.4) | 15 | 0.7 | 0.6 (0.0, 0.2) | 36 | 1.7 | 1.5 (1.1, 2.1) |
| Spoiled | 0 | 0.0 | 0.0 | 2 | 0.1 | 0.1 (0.5, 1.2) | 1 | 0.0 | 0.0 (0.4, 1.0) | 3 | 0.1 | 0.1 (0.0, 0.4) |

| | How would you describe your current level of morale? | | |
|---|---|---|---|
| Very low | 352 | 16.2 | 15.6 (14.1, 17.2) |
| Low | 843 | 38.7 | 38.8 (36.7, 40.9) |
| Neither low nor high | 664 | 30.5 | 30.8 (28.8, 32.8) |
| High | 276 | 12.7 | 12.9 (11.6, 14.4) |
| Very high | 29 | 1.3 | 1.3 (0.9, 1.8) |
| Missing | 11 | 0.5 | 0.5 (0.1, 0.5) |
| Spoiled | 4 | 0.2 | 0.2 (0.3, 0.9) |



For peer review only

For peer review only - http://bmjopen.bmj.com/site/about/guidelines.xhtml

MALE

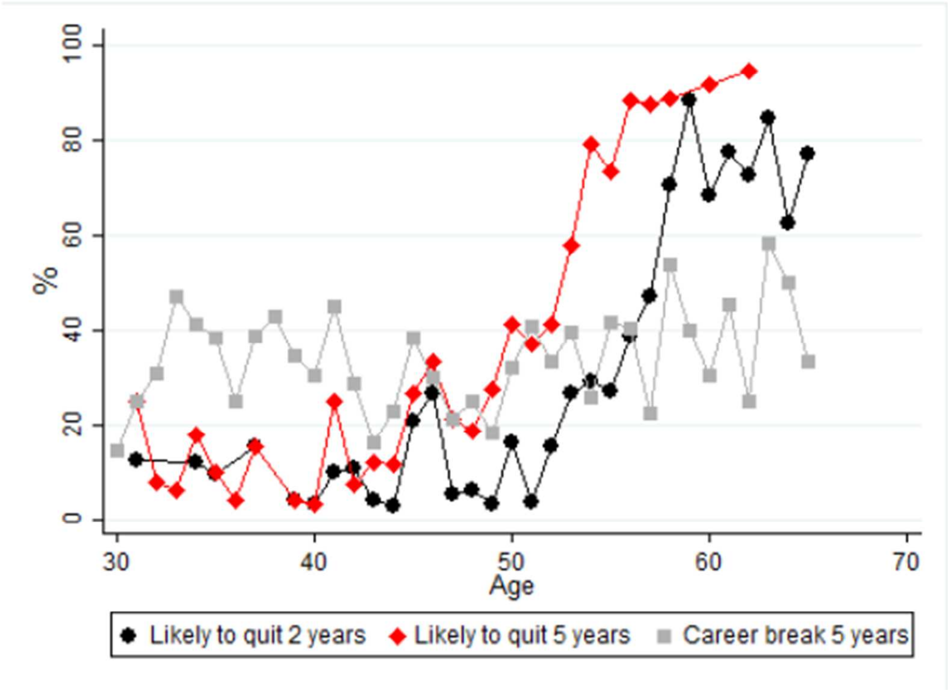


FEMALE

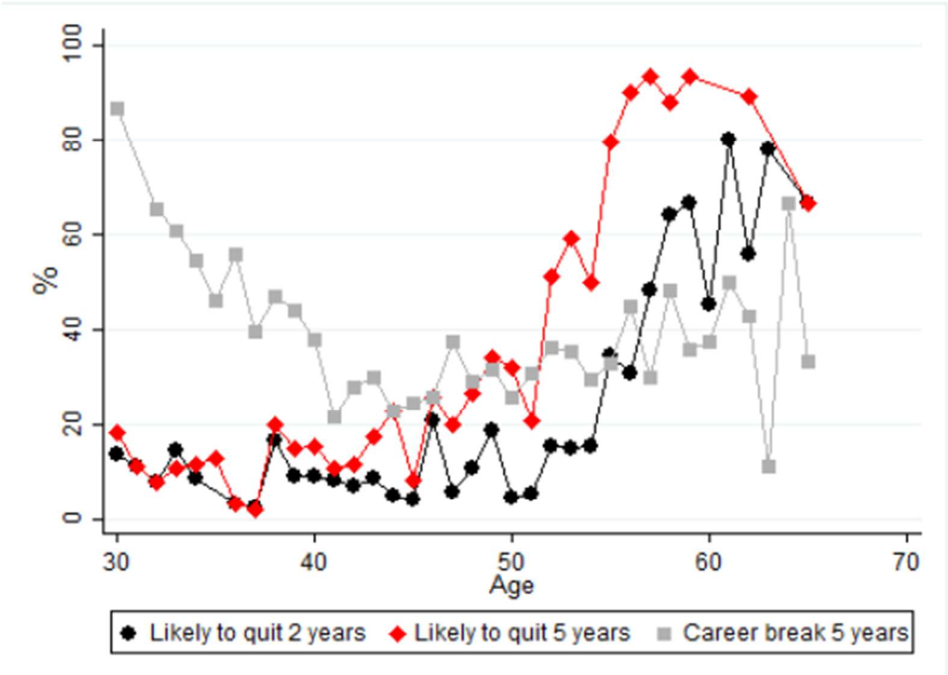


**Figure 1 Career intentions to quit direct patient care by gender and age**



For peer review only - http://bmjopen.bmj.com/site/about/guidelines.xhtml

Age was a very strong predictor of reported intention to leave direct patient care, a finding which persisted after adjustment (for example 60-69 vs. 40-49 year olds OR=194.4 95% CI 84.3, 448.3 for leaving patient care in five years). Age was also a strong predictor of the intention to reduce hours in the adjusted model, with older GPs more likely to report this intention than younger GPs. Female GPs, GPs aged under 40 years, and locum GPs were most likely to report the intention to take a career break after adjustment for other factors. A model including an interaction between age and gender showed that the effect of under 40 year olds being more likely to report intentions to take a career break was strongest in female GPs, consistent with the pattern shown in Figure 1 (results not shown).

Self-reported morale was a strong predictor of all four outcomes shown in Tables 2 and 3. In the unadjusted analysis a 'U-shaped' relationship is seen for all four quitting outcomes, with those with 'very high' morale more likely to report intentions to leave direct patient care/reduce hours/take a career break than those with 'high' or 'neither low nor high' morale (and in the case of leaving patient care in two years, more likely than any other morale category), but less likely to intend to leave/reduce hours/take a career break than those with 'very low' morale. This U-shaped relationship largely disappears however after adjustment, and it is those GPs with 'very low' morale who are most likely to report intending  to leave direct patient care, reduce hours in patient care, or to take a career break. Moreover, the change in odds between 'low' and 'very low' morale is particularly strong. Table 5 shows the results of the logistic regression analysis modelling of factors associated with low or very low morale. Only age and practice role show any evidence of being associated with reported morale (p<0.001 for both). Those aged 50-54 years old are most likely to report low morale. In respect of role, GP partners are the most likely to report low morale, followed by salaried GPs, and with locums and other GPs the least likely groups to report low morale.

Supplementary analyses exploring the contribution of current working patterns to any association with intentions to reduce hours spent in direct patient care did explain some of the difference between genders. However, even after adjustment for current working patterns, female GPs were still substantially less likely to report intentions to reduce hours spent in direct patient care than their male counterparts (not shown).



For peer review only - http://bmjopen.bmj.com/site/about/guidelines.xhtml

**Table 3 Unadjusted associations between career intentions and GP attributes**

| | | How likely is it that you will permanently leave direct patient care within the next 2 years? | | How likely is it that you will permanently leave direct patient care within the next 5 years? | | How likely is it that you will reduce your weekly average hours spent in direct patient care within the next 5 years? | | How likely is it that you will take a career break (or another career break) within the next 5 years? | |
|---|---|---|---|---|---|---|---|---|---|
| | | OR† (95% CI) | p | OR† (95% CI) | p | OR† (95% CI) | p | OR† (95% CI) | p |
| Gender | Male | Reference | <0.001 | Reference | <0.001 | Reference | <0.001 | Reference | 0.046 |
| | Female | 0.51 (0.41, 0.63) | | 0.55 (0.46, 0.66) | | 0.48 (0.41, 0.58) | | 1.20 (1.00, 1.44) | |
| Age | <40 | 0.75 (0.48, 1.16) | <0.001 | 0.49 (0.34, 0.70) | <0.001 | 1.24 (0.98, 1.56) | <0.001 | 2.34 (1.83, 2.98) | <0.001 |
| | 40-49 | Reference | | Reference | | Reference | | Reference | |
| | 50-54 | 1.65 (1.12, 2.43) | | 4.00 (3.02, 5.28) | | 2.04 (1.58, 2.63) | | 1.24 (0.95, 1.63) | |
| | 55-59 | 9.32 (6.72, 12.92) | | 30.04 (21.04, 42.90) | | 5.00 (3.74, 6.69) | | 1.59 (1.22, 2.08) | |
| | 60-69 | 22.22 (14.43, 34.22) | | 91.60 (41.88, 200.36) | | 7.38 (4.58, 11.88) | | 1.61 (1.11, 2.35) | |
| | 70+ | 45.86 (12.84, 163.84) | | * | | 3.86 (1.23, 12.10) | | 1.55 (0.56, 4.32) | |
| Country of qualification | UK/Ireland | Reference | 0.450 | Reference | 0.132 | Reference | 0.921 | Reference | 0.470 |
| | Europe | 0.56 (0.27, 1.14) | | 0.60 (0.35, 1.02) | | 0.85 (0.52, 1.39) | | 0.65 (0.37, 1.12) | |
| | South Asia | 0.88 (0.29, 2.66) | | 0.43 (0.14, 1.32) | | 1.10 (0.45, 2.70) | | 0.95 (0.38, 2.39) | |
| | Other | 1.05 (0.47, 2.32) | | 0.98 (0.51, 1.90) | | 0.95 (0.50, 1.80) | | 0.88 (0.45, 1.73) | |
| Ethnic group | White | Reference | 0.300 | Reference | 0.032 | Reference | 0.581 | Reference | 0.610 |
| | Mixed | * | | 0.60 (0.26, 1.36) | | 0.74 (0.35, 1.57) | | 1.57 (0.74, 3.31) | |
| | Asian | 0.87 (0.48, 1.57) | | 0.43 (0.25, 0.76) | | 1.27 (0.78, 2.05) | | 1.00 (0.61, 1.63) | |
| | Black | 3.59 (0.89, 14.41) | | 1.49 (0.37, 5.99) | | 2.23 (0.45, 11.07) | | 0.60 (0.12, 2.99) | |
| | Other | 0.77 (0.22, 2.69) | | 0.68 (0.24, 1.96) | | 1.36 (0.50, 3.70) | | 1.61 (0.62, 4.18) | |
| Role/ Position | GP Partner | Reference | <0.001 | Reference | <0.001 | Reference | <0.001 | Reference | <0.001 |
| | Salaried GP | 0.61 (0.46, 0.83) | | 0.43 (0.34, 0.55) | | 0.63 (0.51, 0.78) | | 1.25 (1.00, 1.56) | |
| | Locum GP | 1.58 (1.18, 2.11) | | 1.08 (0.82, 1.41) | | 1.34 (1.02, 1.76) | | 1.72 (1.32, 2.24) | |
| | Other | 1.12 (0.61, 2.08) | | 0.72 (0.40, 1.26) | | 0.79 (0.47, 1.34) | | 0.85 (0.48, 1.52) | |
| Reported morale | Very low | 0.99 (0.45, 2.17) | <0.001 | 1.85 (0.81, 4.19) | <0.001 | 2.58 (1.19, 5.59) | <0.001 | 2.24 (1.01, 4.95) | <0.001 |
| | Low | 0.42 (0.19, 0.91) | | 0.78 (0.35, 1.75) | | 1.31 (0.62, 2.77) | | 1.14 (0.52, 2.49) | |
| | Neither low/high | 0.30 (0.14, 0.65) | | 0.62 (0.28, 1.40) | | 0.82 (0.39, 1.73) | | 0.81 (0.37, 1.78) | |
| | High | 0.40 (0.18, 0.90) | | 0.62 (0.27, 1.42) | | 0.54 (0.25, 1.16) | | 0.55 (0.24, 1.24) | |
| | Very high | Reference | | Reference | | Reference | | Reference | |

† ORs>1 indicate category is more likely to leave direct patient care/reduce hours/take a career break than the reference category

* This group has been excluded from the analysis due to perfect prediction, i.e. all over 70 year olds reported being likely to leave direct patient in 5 years and no mixed ethnicity GPs reported being likely to leave direct patient care in 2 years



For peer review only

**Table 4 Adjusted associations between career intentions and GP attributes. Adjustment was made for all factors shown in table.**

| | | How likely is it that you will permanently leave direct patient care within the next 2 years? | | How likely is it that you will permanently leave direct patient care within the next 5 years? | | How likely is it that you will reduce your weekly average hours spent in direct patient care within the next 5 years? | | How likely is it that you will take a career break (or another career break) within the next 5 years? | |
|---|---|---|---|---|---|---|---|---|---|
| | | OR† (95% CI) | p | OR† (95% CI) | p | OR† (95% CI) | p | OR† (95% CI) | p |
| Gender | Male | Reference | 0.181 | Reference | 0.810 | Reference | <0.001 | Reference | 0.019 |
| | Female | 0.83 (0.64, 1.09) | | 1.03 (0.80, 1.33) | | 0.58 (0.47, 0.71) | | 1.27 (1.04, 1.55) | |
| Age | <40 | 0.89 (0.56, 1.41) | <0.001 | 0.58 (0.40, 0.85) | <0.001 | 1.47 (1.14, 1.90) | <0.001 | 2.63 (2.02, 3.42) | <0.001 |
| | 40-49 | Reference | | Reference | | Reference | | Reference | |
| | 50-54 | 1.70 (1.14, 2.55) | | 4.73 (3.49, 6.41) | | 2.11 (1.61, 2.77) | | 1.31 (0.98, 1.73) | |
| | 55-59 | 11.69 (8.18, 16.72) | | 44.15 (29.77, 65.48) | | 5.62 (4.12, 7.66) | | 1.79 (1.35, 2.38) | |
| | 60-69 | 36.87 (22.53, 60.34) | | 194.43 (84.32, 448.34) | | 9.58 (5.76, 15.95) | | 2.03 (1.35, 3.04) | |
| | 70+ | 110.61 (25.85, 473.26) | | * | | 4.93 (1.47, 16.55) | | 1.83 (0.61, 5.48) | |
| Country of qualification | UK/Ireland | Reference | 0.193 | Reference | 0.048 | Reference | 0.829 | Reference | 0.738 |
| | Europe | 0.60 (0.27, 1.37) | | 0.48 (0.23, 0.99) | | 0.98 (0.57, 1.70) | | 0.73 (0.41, 1.29) | |
| | South Asia | 0.21 (0.04, 1.15) | | 0.13 (0.02, 0.97) | | 0.61 (0.19, 1.96) | | 0.98 (0.31, 3.05) | |
| | Other | 0.83 (0.30, 2.31) | | 0.94 (0.39, 2.25) | | 0.83 (0.40, 1.71) | | 1.08 (0.53, 2.22) | |
| Ethnic group | White | Reference | 0.109 | Reference | 0.142 | Reference | 0.209 | Reference | 0.483 |
| | Mixed | * | | 2.84 (1.15, 7.04) | | 1.32 (0.60, 2.92) | | 1.74 (0.79, 3.81) | |
| | Asian | 1.58 (0.70, 3.54) | | 0.84 (0.38, 1.86) | | 1.89 (1.04, 3.46) | | 0.87 (0.48, 1.57) | |
| | Black | 6.07 (1.00, 36.99) | | 2.26 (0.29, 17.71) | | 2.89 (0.48, 17.28) | | 0.54 (0.10, 2.87) | |
| | Other | 0.45 (0.10, 2.16) | | 0.45 (0.08, 2.43) | | 1.17 (0.39, 3.45) | | 1.57 (0.56, 4.41) | |
| Role/ Position | GP Partner | Reference | 0.177 | Reference | 0.153 | Reference | 0.005 | Reference | <0.001 |
| | Salaried GP | 1.10 (0.76, 1.59) | | 0.81 (0.58, 1.14) | | 0.97 (0.75, 1.24) | | 1.15 (0.89, 1.47) | |
| | Locum GP | 1.55 (1.04, 2.30) | | 1.37 (0.91, 2.07) | | 1.74 (1.26, 2.39) | | 2.09 (1.55, 2.81) | |
| | Other | 0.91 (0.39, 2.15) | | 0.73 (0.29, 1.86) | | 1.19 (0.64, 2.20) | | 1.15 (0.62, 2.13) | |
| Reported morale | Very low | 5.77 (1.96, 17.00) | <0.001 | 13.07 (3.72, 45.88) | <0.001 | 6.74 (2.76, 16.47) | <0.001 | 3.49 (1.49, 8.14) | <0.001 |
| | Low | 1.87 (0.65, 5.38) | | 3.89 (1.13, 13.34) | | 3.27 (1.38, 7.74) | | 1.50 (0.66, 3.45) | |
| | Neither low/high | 0.86 (0.30, 2.47) | | 2.15 (0.63, 7.39) | | 1.75 (0.74, 4.14) | | 0.93 (0.40, 2.12) | |
| | High | 0.80 (0.27, 2.39) | | 1.29 (0.36, 4.60) | | 0.86 (0.36, 2.08) | | 0.59 (0.25, 1.40) | |
| | Very high | Reference | | Reference | | Reference | | Reference | |

† ORs>1 indicate category is more likely to leave direct patient care/reduce hours/take a career break than the reference category

* This group has been excluded from the analysis due to perfect prediction, i.e. all over 70 year olds reported being likely to leave direct patient in 5 years and no mixed ethnicity GPs reported being likely to leave direct patient care in 2 years



For peer review only - http://bmjopen.bmj.com/site/about/guidelines.xhtml

**Table 5 Unadjusted and adjusted associations between self-reported low or very low morale and GP attributes. Adjustment was made for all factors shown in table.**

| | | Unadjusted | | Adjusted | |
|---|---|---|---|---|---|
| | | OR† (95% CI) | p | OR† (95% CI) | p |
| Gender | Male | Reference | 0.134 | Reference | 0.231 |
| | Female | 0.87 (0.72 ,0.72) | | 0.90 (0.76 ,0.76) | |
| Age | <40 | 0.73 (0.57 ,0.57) | <0.001 | 0.69 (0.55 ,0.55) | <0.001 |
| | 40-49 | Reference | | Reference | |
| | 50-54 | 1.06 (0.81 ,0.81) | | 1.10 (0.85 ,0.85) | |
| | 55-59 | 0.85 (0.66 ,0.66) | | 0.92 (0.71 ,0.71) | |
| | 60-69 | 0.38 (0.26 ,0.26) | | 0.33 (0.23 ,0.23) | |
| | 70+ | 0.07 (0.01 ,0.01) | | 0.04 (0.01 ,0.01) | |
| Country of qualification | UK/Ireland | Reference | 0.157 | Reference | 0.194 |
| | Europe | 0.74 (0.45 ,0.45) | | 0.73 (0.45 ,0.45) | |
| | South Asia | 0.97 (0.32 ,0.32) | | 0.97 (0.40 ,0.40) | |
| | Other | 0.50 (0.25 ,0.25) | | 0.55 (0.29 ,0.29) | |
| Ethnic group | White | Reference | 0.534 | Reference | 0.721 |
| | Mixed | 0.82 (0.38 ,0.38) | | 0.82 (0.39 ,0.39) | |
| | Asian | 1.59 (0.89 ,0.89) | | 1.31 (0.81 ,0.81) | |
| | Black | 1.64 (0.37 ,0.37) | | 0.82 (0.20 ,0.20) | |
| | Other | 1.01 (0.36 ,0.36) | | 0.73 (0.28 ,0.28) | |
| Role/ Position | GP Partner | Reference | <0.001 | Reference | <0.001 |
| | Salaried GP | 0.75 (0.59 ,0.59) | | 0.67 (0.54 ,0.54) | |
| | Locum GP | 0.40 (0.30 ,0.30) | | 0.33 (0.25 ,0.25) | |
| | Other | 0.22 (0.12 ,0.12) | | 0.20 (0.11 ,0.11) | |

† ORs>1 indicate category is more likely to report low or very low morale than the reference category

For peer review only

# DISCUSSION

## Main findings

A high proportion of GPs – around two in every five – currently working within direct patient care in South West England reported an intention to permanently quit direct patient care within the next five years, this being one in five within the next two years. Further depletion of the GP workforce in this region of England through reduction of weekly average hours or through taking a career break within the next five years also emerged as an impending risk. Overall, seven out of every ten GPs in this region reported a career intention which, if implemented, would adversely impact the GP workforce capacity in South West England through GPs leaving direct patient care, reducing hours spent in direct patient care, or by taking a career break within the next five years.

Older age is highly predictive of a GP's intentions to permanently quit direct patient care and to reduce hours. The intention to quit was independent of the GP's role (partner, salaried or locum) and gender. The gender differences observed amongst younger GPs in respect of intended career breaks is a particular issue where, as currently exists, 69% of the GP trainee workforce is female(17), and where a substantial proportion of younger women returning to clinical care do so on the basis of reduced hours(1), and where doctors who wish to return to clinical practice may face significant bureaucratic obstacles in doing so(18). The decision to reduce hours or to take a career break varied with employment status, with locum GPs being most likely to report intentions to reduce hours or to take a career break. In a situation where GPs who are not partners in a practice do in fact leave direct patient care, the system of care appears vulnerable, with added strain likely to be placed on GPs who are partners in their practice.

A key issue is whether the intention to leave is translated into reality, and few studies have explored this important question. However, Hann et al (19) explored this question amongst nearly 1200 UK GPs followed up over five years, and identified a strong relationship between a stated intention to leave patient care and actually doing so, reflected in a 4.5-fold difference in odds of leaving between those with no stated intention to leave patient care when compared with those reporting a high intention to do so.

The majority of responding GPs in our study reported low morale, with less than 15% reporting high morale. GP morale was identified as an important predictor of future career intentions, particularly when morale was very low.



For peer review only - http://bmjopen.bmj.com/site/about/guidelines.xhtml

**Strengths and limitations of the study**

By ensuring alignment of wording with other surveys of similar intent allows the opportunity for comparing and contrasting of results. Recent high quality surveys support our estimate of the proportion of GPs intending to leave general practice within the next five years (14, 20-22). Unlike most other similar surveys which tend to survey samples of GPs(7, 23), we undertook a census survey of all GPs currently eligible to provide patient care across a large area, providing a cross-sectional overview of the quit intentions of all GPs in South West England. The response rate was high (67%), reflecting both rigorous planning and implementation of the survey, and interest among GPs in the subject matter of workforce challenges. Further, we have reduced the potential for non-response bias by weighting our estimates to account for differential response rates by age, gender and practice role. The overall programme of work, of which this survey is one element, includes the planned development of a predictive risk modelling tool to enumerate risk of future workforce undersupply at local levels, thus offering the potential of providing data that will be of value to healthcare planners and policy makers. In addition, our study relates to a key current area of national interest and concern.

The study has a number of limitations. The survey was conducted amongst GPs in a single region of England which may not be entirely typical of England as a whole, for example in respect of population-mix and practice setting(24). The region is often informally regarded as having desirable living and working conditions(25). It seems likely that other regions of England, with more inner-city areas with higher levels of deprivation, are likely to face even greater challenges in respect of the GP workforce than in the findings we have presented here. A further limitation is that the research was undertaken within a rapidly changing GP workforce policy environment, for example, the implementation in 2015-16 of a 10-point plan agreed between authoritative UK health, governmental, and professional bodies concerned about GP workforce issues(26). Finally, this survey was cross-sectional, rather than longitudinal in design, and so we are unable to report on the actual translation of GPs' reported career intentions into actual career decisions, although previous work has demonstrated intention to quit to be a strong predictor of actually leaving(19).

**Implications for research**

Our survey is part of a multi-method suite of work commissioned by the NHS, seeking to inform evidence-based policies and strategies in a key area of national concern, and which will involve stakeholder consultations with policy-makers who have oversight of GP workforce planning(16). Future research should include assessment of the predictive validity of GPs' reported career intentions by longitudinal follow-up of GPs, investigation of how reducing hours or better flexible



For peer review only - http://bmjopen.bmj.com/site/about/guidelines.xhtml

working arrangements may influence GP retention, and undertaking research exploring the potential for extended clinical roles amongst important allied healthcare professionals in the clinical workforce. Such groups might include nurses, physiotherapists and pharmacists, and developing such extended roles for these professionals within the primary care team may offer a rapid and potentially effective contribution to the alleviation of current GP workforce pressures. Research into the determinants and impact of working reduced hours should also encompass other aspects of flexible working arrangements in general practice, and how such arrangements may improve job satisfaction and work-life balance, and thereby potentially increase GP retention in the longer term. This work also provides insight into which groups of GPs might best be targeted with interventions aiming to improve workforce retention.

**Implications for policy and practice**

Concerns regarding the GP workforce are now recognised widely by authoritative bodies in the UK, leading to recent policy statements and initiatives(26) and to the establishment of a Primary Care Workforce Commission by Health Education England(4). Our research can inform this policy agenda by providing an estimate of the likely proportion of South West GPs intending to leave direct patient care within the next five years through permanently quitting patient care or by taking a career break. Our findings are likely to be of relevance in other areas of England that may face even greater challenges in respect of the GP workforce. Retaining the direct patient care capacity of established GPs represents an immediate challenge for healthcare planners. Recognising and addressing issues of low GP morale is an area of importance, and addressing the issue may not only improve how GPs feel about their work, but also potentially be associated with improvements in patients' experience of the care received(27). In the face of problems recruiting to general practice(28) and the wider workforce problems, the UK government recently announced an increase in the number of training places for GPs(26) and an increase in the number of medical training places in the UK(29). But these medium- to long-term solutions do not address the immediate workforce crisis. In contrast, policy initiatives to support the retention of the existing GP workforce, the rapid development of and support for new models of care, and the implementation of policies aimed at alleviating workload pressures for GPs, are potential short- to medium-term solutions which need to be urgently considered. Developing and implementing relevant policies and strategies to achieve those ambitions will be important for the alleviation of the immediate pressures faced by the GP workforce and the populations of patients they serve.

**Conclusions**



For peer review only - http://bmjopen.bmj.com/site/about/guidelines.xhtml

Healthcare in England faces imminent challenges in respect of GP workforce capacity. This survey identifies the magnitude of the problem in South West England and highlights the important role of GP morale as one important factor contributing to that challenge. Acknowledgement of the magnitude of the problems is urgently required, along with implementation and monitoring of relevant policy and strategy. Failure to do so will risk serious adverse effects on the capacity and ability of the NHS to provide effective primary care to the UK population.

For peer review only



For peer review only - http://bmjopen.bmj.com/site/about/guidelines.xhtml

**What is already known on this topic**

The UK faces serious challenges over the next few years in respect of the capacity of the GP workforce. The UK government has instituted a range of measures to train more doctors, and to attract more doctors into general practice. Previous research has examined the career intentions of samples of UK GPs.

**What this study adds**

A census survey of GPs in South West England reveals serious and imminent potential problems in respect of the capacity of the GP workforce in this region of England – an area often judged as 'desirable' and with lower than average socio-economic deprivation. Less than 1 in 5 of the GPs participating in this survey reported high morale and many GPs, especially those over the age of 50 years, reported considering permanently leaving direct patient care.

**Acknowledgements**

We are grateful to Dr Nigel Acheson and Dr Liz Thomas (NHS England) for their support with the conduct of this research, to all of the GP participants who contributed survey responses, to the PPI group chaired by Dr Jo Welsman who support the wider programme of work of which the survey is part, to Alex Aylward, patient representative, who commented on survey design, and to Dr Jo Roberts, (South West Academic Health Science Network and South Devon CCG) and other members of the study advisory group.

**Contributors**

EF contributed to the study design, data collection, analysis and writing of the paper. JC contributed to study design, analysis and writing of the paper. RA, SR, CS, SD, AS and FW contributed to the study design and writing of the paper. GA contributed to analysis and writing of the paper. All authors read and approved the final paper. JC is the guarantor of the paper.

**Funding**

The project was funded by the National Institute for Health Research, Health Service and Delivery Research programme (project 14/196/02). The views and opinions expressed herein are those of the authors and do not necessarily reflect those of the Health Service and Delivery Research programme, the National Institute for Health Research, the National Health Service, or the Department of Health.

**Ethical approval**



For peer review only - http://bmjopen.bmj.com/site/about/guidelines.xhtml

This study was approved by the University of Exeter Medical School Research Ethics Committee (15/11/085).

**Competing interests**

All authors have completed the ICMJE uniform disclosure form at www.icmje.org/coi_disclosure.pdf (available on request from the corresponding author) and declare: grants from the National Institute for Health Research (Health Service and Delivery Research programme) during the conduct of the study. SD's position is partly supported by the National Institute for Health Research (NIHR) Collaboration for Leadership in Applied Health Research and Care South West Peninsula at the Royal Devon and Exeter NHS Foundation Trust. The views expressed are those of the author(s) and not necessarily those of the NHS, the NIHR or the Department of Health.

**Data sharing**

Requests for statistical code and dataset can be made to the corresponding author at john.campbell@exeter.ac.uk. The dataset will be made available via a publicly accessible repository on publication.

All authors, external and internal, had full access to all of the data (including statistical reports and tables) in the study and can take responsibility for the integrity of the data and the accuracy of the data analysis.

**Transparency**

The lead author (EF) and corresponding author (JC) affirm that the manuscript is an honest, accurate, and transparent account of the study being reported, and that no aspects of the study have been omitted.

The Corresponding Author has the right to grant on behalf of all authors and does grant on behalf of all authors, a worldwide licence (http://www.bmj.com/sites/default/files/BMJ%20Author%20Licence%20March%202013.doc) to the Publishers and its licensees in perpetuity, in all forms, formats and media (whether known now or created in the future), to i) publish, reproduce, distribute, display and store the Contribution, ii) translate the Contribution into other languages, create adaptations, reprints, include within collections and create summaries, extracts and/or, abstracts of the Contribution and convert or allow conversion into any format including without limitation audio, iii) create any other derivative work(s) based in whole or part on the on the Contribution, iv) to exploit all subsidiary rights to exploit all subsidiary rights that currently exist or as may exist in the future in the Contribution, v)



For peer review only - http://bmjopen.bmj.com/site/about/guidelines.xhtml

the inclusion of electronic links from the Contribution to third party material where-ever it may be located; and, vi) licence any third party to do any or all of the above. All research articles will be made available on an Open Access basis (with authors being asked to pay an open access fee—see http://www.bmj.com/about-bmj/resources-authors/forms-policies-and-checklists/copyright-open-access-and-permission-reuse). The terms of such Open Access shall be governed by a Creative Commons licence—details as to which Creative Commons licence will apply to the research article are set out in our worldwide licence referred to above.

## References

1. Centre for Workforce Intelligence. In-depth review of the general practitioner workforce: final report. London; 2014.
2. Hobbs FR, Bankhead C, Mukhtar T, Stevens S, Perera-Salazar R, Holt T, et al. Clinical workload in UK primary care: a retrospective analysis of 100 million consultations in England, 2007–14. The Lancet. 2016;387(10035):2323-30.
3. Berwick DM. A transatlantic review of the NHS at 60. British Medical Journal,. 2008;337(7663):212-4.
4. Health Education England (HEE) Commission on GP workforce. Securing the Future GP Workforce - Delivering the Mandate on GP Expansion. London: Health Education England; 2014.
5. British Medical Association. BMA cohort study of 2006 medical graduates. Eighth report. London; 2014.
6. Sansom A, Calitri R, Carter M, Campbell J. Understanding quit decisions in primary care: a qualitative study of older GPs. BMJ Open. 2016;6(2).
7. Hann M, McDonald J, Checkland K, Coleman A, Gravelle H, Sibbald B, et al. Seventh national GP worklife survey. Manchester: University of Manchester; 2013.
8. NHS Digital. General and personal medical services, England – 2004–2014 as at 30 September Leeds: NHS Digital; 2015 [Available from: http://content.digital.nhs.uk/catalogue/PUB16934.
9. Dayan M, Arora S, Rosen R, Curry N. Is general practice in crisis? London: Nuffield Trust; 2014.
10. Baird B, Charles,A., Honeyman, M., Maguire, D., Das, P. Understanding pressures in general practice London: King's Fund; 2016 [updated 1/12/2016. Available from: https://www.kingsfund.org.uk/sites/files/kf/field/field_publication_file/Understanding-GP-pressures-Kings-Fund-May-2016.pdf.
11. Pulse. RCGP petitions Government to create new 'medical assistant' role to relieve admin pressures on GPs 2014 [Available from: http://www.pulsetoday.co.uk/20008118.article#.VD_Iv01wbcs.
12. NHS. Five year forward view. London: NHS England; 2014.
13. The King's Fund and Nuffield Trust. Securing the Future of General Practice: New Models of Primary Care London2013 [Available from: http://www.nuffieldtrust.org.uk/publications/securing-future-general-practice.
14. Campbell J, Calitri, R., Sansom, A. Retaining the experienced GP workforce in direct patient care (ReGROUP): Final Report. Exeter: University of Exeter; 2015.
15. NHS England National Performers List London2016 [Available from: https://www.performer.england.nhs.uk/.
16. Primary Care Research Group UoEMS. The changing general practitioner worforce: the development of policies and strategies aimed at retaining experienced GPs and those taking a career break in direct patient care: ReGROUP project 2016 [Available from: http://medicine.exeter.ac.uk/research/healthresearch/regroup/.



For peer review only - http://bmjopen.bmj.com/site/about/guidelines.xhtml

17. General Medical Council. The state of medical education and practice in the UK. London; 2015.
18. Royal College of General Practitioners. Thousands of doctors prevented from returning to GP workforce by red tape London: RCGP; 2014 [Available from: http://www.rcgp.org.uk/news/2014/august/thousands-of-doctors-prevented-from-returning-to-gp-workforce-by-red-tape.aspx.
19. Hann M, Reeves D, Sibbald B. Relationships between job satisfaction, intentions to leave family practice and actually leaving among family physicians in England. European journal of public health. 2011;21(4):499-503.
20. Gibson J, Checkland K, Coleman A, Hann M, McCall R, Spooner S, et al. Eighth national GP worklife survey. Manchester; 2015.
21. Dale J, Potter R, Owen K, Parsons N, Realpe A, Leach J. Retaining the general practitioner workforce in England: what matters to GPs? A cross-sectional study. BMC family practice. 2015;16.
22. Martin SD, E. Gershlick, B. Under pressure: What the Commonwealth Fund's 2015 international survey of general practitioners means for the UK. London: The Health Foundation; 2016.
23. British Medical Association. BMA quarterly tracker survey: current views from across the medical profession. Quarter 3: July/August 2014. London; 2014.
24. UK Government. English Indices of Deprivation 2015 2015 [Available from: https://www.gov.uk/government/statistics/english-indices-of-deprivation-2015.
25. wikipedia. South West England 2016 [Available from: https://en.wikipedia.org/wiki/South_West_England#Deprivation.
26. NHS England. GP five year forward view. London: Department of Health; 2016.
27. King's College London: National Nursing Research Unit. Does NHS staff wellbeing affect patient experience of care? Policy plus evidence, issues and opinions in healthcare. 2013;May 2013(39).
28. Rimmer A. A third of GP training posts are vacant after first recruitment round London: BMJ; 2015 [Available from: http://careers.bmj.com/careers/advice/A_third_of_GP_training_posts_are_vacant_after_first_recruitment_round.
29. Rt Hon J Hunt MP Secretary of State for Health. Up to 1,500 extra medical training places announced London: UK Department of Health; 2016 [Available from: https://www.gov.uk/government/news/up-to-1500-extra-medical-training-places-announced.

For peer review only

MALE

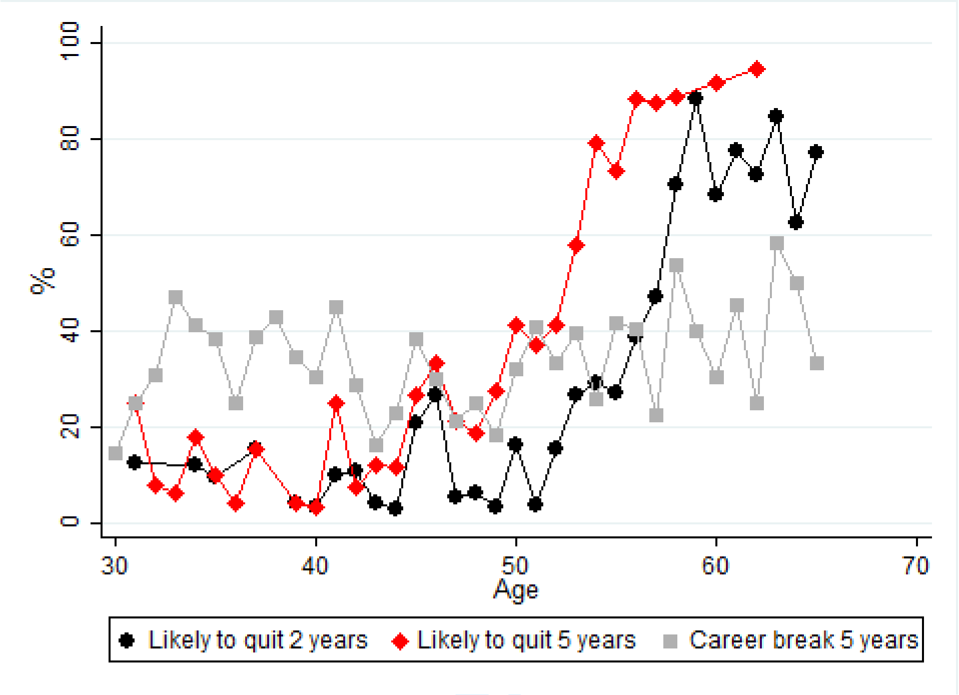
%
0
20
40
60
80
100
30
40
50
60
70
Age
Likely to quit 2 years
Likely to quit 5 years
Career break 5 years

FEMALE

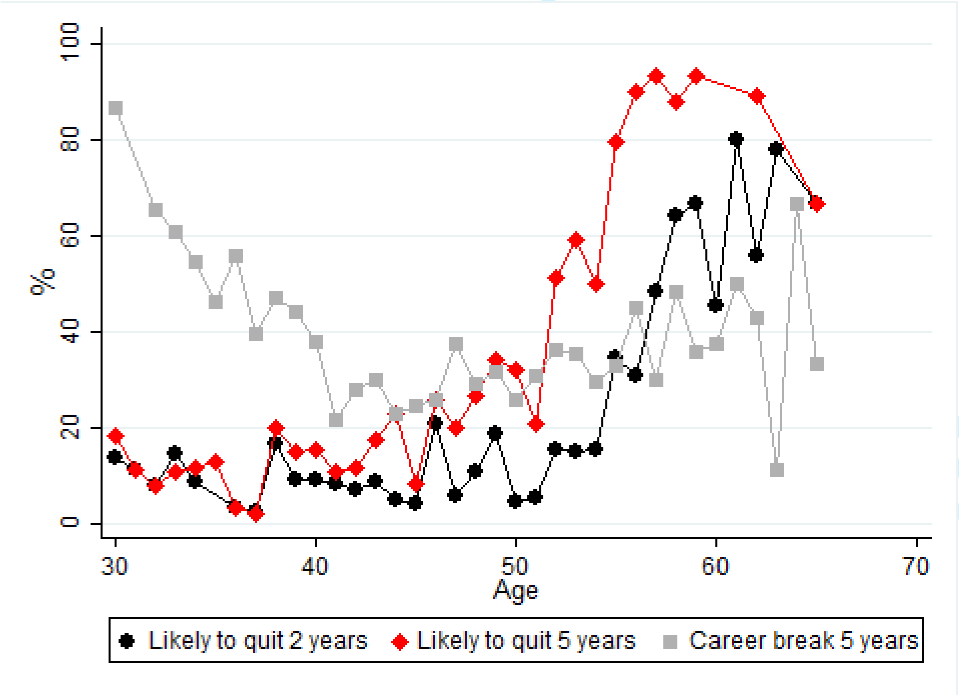
%
0
20
40
60
80
100
30
40
50
60
70
Age
Likely to quit 2 years
Likely to quit 5 years
Career break 5 years

**Supplementary Online Material Table 1. Sample characteristics**

| Characteristic (N/3370) | Responded n (%) |
|---|---|
| **Gender (3370)** | |
| Male | 1051 (67) |
| Female | 1197 (66) |
| **Age (years; 3370)** | |
| Under 40 | 500 (54) |
| 40-49 | 738 (68) |
| 50-54 | 391 (72) |
| 55-59 | 409 (76) |
| 60-69 | 192 (74) |
| 70 and over | 18 (75) |
| **Employment Status (3370)** | |
| GP provider | 1335 (71) |
| GP salaried | 394 (57) |
| Non-principal/locum | 519 (64) |
| **Survey sent by email (3370)** | |
| Yes | 1304 (71) |
| No | 944 (62) |
| **Postal address used (3370)** | |
| Practice address | 1744 (67) |
| Home address | 504 (64) |

For peer review only

Supplementary Online Material - Questionnaire

National Institute for Health Research | University of Exeter Medical School | University of Bristol | University of Exeter Business School | Centre for Workforce Intelligence

# SUPPORTING THE GP WORKFORCE - ReGROUP QUESTIONNAIRE

**This questionnaire is part of a research study examining the issue of the GP workforce. Please help by completing the questionnaire. Please answer the questions below by ticking one box for each question and printing text where required in block capitals. We will keep your answers completely confidential.**

**If you would prefer to complete the survey online, please go to** ex.ac.uk/GP_Workforce

**Reference** 1234567890

**'Direct patient care' includes time spent in all routine general practice activities (in the UK), including consultations, telephone, clinical and practice administration, prescribing and referral.**

## GP WORKFORCE QUESTIONS

Q.1 **Please tell us which of the following scenarios applies to you?**

☐ I am currently working in direct patient care
☐ I am currently on a career break (i.e. a break of 6 months or more for maternity/paternity leave or personal/professional development, including doing medical work abroad)
☐ I have permanently left direct patient care within the last 5 years.................................**Go to Q.9**
☐ None of the above...........................**Go to Q.9**

Q.2 **How likely is it that you will permanently leave direct patient care within the next 2 years?**

☐ Very Likely
☐ Likely
☐ Unlikely
☐ Very unlikely

Q.3 **How likely is it that you will permanently leave direct patient care within the next 5 years?**

☐ Very Likely
☐ Likely
☐ Unlikely
☐ Very unlikely

Q.4 **Overall, have you reduced your weekly average hours spent in direct patient care within the last 5 years?**

☐ No
☐ Yes

Q.5 **How likely is it that you will reduce your weekly average hours spent in direct patient care within the next 5 years?**

☐ Very Likely
☐ Likely
☐ Unlikely

Q.6 **How likely is it that you will take a career break (or another career break) within the next 5 years?**

☐ Very Likely
☐ Likely
☐ Unlikely
☐ Very unlikely

Q.7 **How would you describe your current level of morale?**

☐ Very Low
☐ Low
☐ Neither low nor high
☐ High
☐ Very high

Q.8 **Have you taken any steps toward changing your work-life balance (e.g. sought pension advice, consulted LMC, BMA etc)?**

☐ No, I am still considering my options
☐ Yes, I am in the process of making this change
☐ Yes, I have made this change

## ABOUT YOU

Q.9 **What is your gender?**

☐ Male
☐ Female
☐ Prefer not to say

Q.10 **What is your age? (to nearest year)**

Q.11 **What year did you qualify in medicine? (YYYY)**

<RESEARCH ID>



**Please turn over** ☞

For peer review only - http://bmjopen.bmj.com/site/about/guidelines.xhtml

☐Very unlikely

Q.12 **What year did you qualify as a GP? (YYYY)**

Q.13 **From what region did you obtain your primary medical qualification?**

☐UK/Ireland
☐Europe (non-UK/Ireland)
☐South Asia
☐Other

Q.14 **What is your ethnic group?**

☐White
☐Mixed/multiple ethnic groups
☐Asian/Asian British
☐Black/African/Caribbean/Black British
☐Other ethnic group
↳ **Please write in:**

Q.15 **In your current/most recent direct patient care role, what is/was your position?**

☐GP Partner
☐Salaried GP
☐Locum GP
☐Other
↳ **Please write in:**

Q.16 **In your current/most recent direct patient role, how many sessions do/did you work in a typical week? (Please consider a session as a block of approximately four hours of clinical time)**

↳ **Please write in:**

**sessions**

Q.17 **When do you work these sessions in a typical week? Please tick the appropriate boxes:**

| | MON | TUE | WED | THU | FRI | SAT | SUN |
|---|---|---|---|---|---|---|---|
| Morning | ☐ | ☐ | ☐ | ☐ | ☐ | ☐ | ☐ |
| Afternoon | ☐ | ☐ | ☐ | ☐ | ☐ | ☐ | ☐ |
| Evening | ☐ | ☐ | ☐ | ☐ | ☐ | ☐ | ☐ |

Q.18 **Do you have a practice that you are primarily affiliated to in your most recent direct patient care role?**

☐No
☐Yes
↳ **If yes, please write in the practice name AND ODS code (e.g. L123456), if known:**

Q.19 **Are you personally involved in the delivery of out of hours direct patient care?**

☐Yes
☐No

## CAN WE TALK TO YOU?

Q.20 **Would you be willing to take part in a confidential interview (45mins) to discuss issues around work-life balance? (You would be reimbursed for time)**

☐Yes
☐No

Q.21 **We plan on developing policies and strategies to help support the experienced GP workforce. Would you agree to be approached by this research team for other research studies that address this agenda?**

☐Yes
☐No

Q.22 **If you would be willing to be interviewed and/or be contacted for other research studies addressing the workforce agenda, please provide:**

***Preferred method of contact*** ⇩

**Your email address:** ☐

______________________________________

**Your telephone number:** ☐

______________________________________

Q.23 **What is your preferred time of day to be contacted?**

☐Morning
☐Lunchtime
☐Afternoon
☐Evening

**On:**
☐Weekdays
☐Weekends

## PRIZE DRAW

Q.24 **Please tick the box below if you would like to be entered into a prize draw to receive one of five Kindles**

☐Yes, please enter me into the prize draw

**THANK YOU FOR YOUR TIME**

**Please return this questionnaire in the reply paid envelope provided (no stamp is needed)**

For peer review only - http://bmjopen.bmj.com/site/about/guidelines.xhtml

BMJ Open

# Quitting patient care and career break intentions among general practitioners in South West England: findings of a census survey of general practitioners.



| | |
|---|---|
| Journal: | *BMJ Open* |
| Manuscript ID | bmjopen-2017-015853.R1 |
| Article Type: | Research |
| Date Submitted by the Author: | 09-Mar-2017 |
| Complete List of Authors: | Fletcher, Emily; University of Exeter, Primary Care Research Group, University of Exeter Medical School<br>Abel, Gary; University of Exeter, Primary Care Research Group, University of Exeter Medical School<br>Anderson, Rob; University of Exeter, Evidence Synthesis & Modelling for Health Improvement, University of Exeter Medical School<br>Richards, Suzanne; University of Exeter Medical School, Primary Care Research Group, University of Exeter Medical School<br>Salisbury, Chris; University of Bristol, Centre for Academic Primary Care, School of Social and Community Medicine<br>Dean, Sarah; University of Exeter, Psychology Applied to Rehabilitation and Health, University of Exeter Medical School<br>Sansom, Anna; University of Exeter Medical School, Primary Care<br>Warren, Fiona; University of Exeter Medical School, Institute of Health Research<br>Campbell, John; University of Exeter, Primary Care Research Group, University of Exeter Medical School |
| <b>Primary Subject Heading</b>: | General practice / Family practice |
| Secondary Subject Heading: | Health services research |
| Keywords: | PRIMARY CARE, GENERAL PRACTICE, WORKFORCE, MORALE, SURVEY, Organisation of health services < HEALTH SERVICES ADMINISTRATION & MANAGEMENT |
| | |



SCHOLARONE™
Manuscripts

# Quitting patient care and career break intentions among general practitioners in South West England: findings of a census survey of general practitioners.

Emily Fletcher *research fellow*[1], Gary Abel *statistician*[1], Rob Anderson *associate professor*[2] Suzanne H Richards *senior lecturer*[1], Chris Salisbury *professor of primary health care*[3], Sarah Dean *associate professor*[4], Anna Sansom *research fellow*[1], Fiona Warren *statistician*[1], John L Campbell *professor of general practice and primary care*[1*]

[1] Primary Care Research Group, University of Exeter Medical School, Exeter, UK

[2] Evidence Synthesis & Modelling for Health Improvement, University of Exeter Medical School, Exeter, UK

[3]Centre for Academic Primary Care, NIHR School for Primary Care Research, School of Social and Community Medicine, University of Bristol, Bristol, UK

[4]Psychology Applied to Rehabilitation and Health, University of Exeter Medical School, Exeter, UK

*Corresponding author:

Professor John Campbell
Primary Care Research Group
University of Exeter Medical School
Smeall Building, St Luke's Campus
Magdalen Road
Exeter
EX1 2LU
john.campbell@exeter.ac.uk

Word count (excludes title page, abstract, references, figures, tables):

Key words: primary care; general practice; workforce; morale; survey; organisation of care



For peer review only - http://bmjopen.bmj.com/site/about/guidelines.xhtml

**ABSTRACT**

**Objective** Given recent concerns regarding general practice (GP) workforce capacity, we aimed to describe GPs' career intentions, especially those which might impact on GP workforce availability over the next 5 years.

**Design** Census survey, conducted between April and May 2016 using postal and online responses, of all GPs on the NHS Performers list and eligible to practise in primary care. Two reminders were used as necessary.

**Setting** South West England (population 3.5 million), a region with low overall socio-economic deprivation.

**Participants** 2248 out of 3370 eligible GPs (67% response rate).

**Main outcome measures** Reported likelihood of permanently leaving or reducing hours spent in direct patient care or of taking a career break within the next five years, and present morale weighted for non-response.

**Results** Responders included 2179 GPs engaged in patient care. Of these, 863 (37% weighted, 95%CI 35%, 39%) reported a high likelihood of quitting direct patient care within the next five years. Overall, 1535 (70% weighted, 95%CI 68%, 72%) respondents reported a career intention that would negatively impact GP workforce capacity over the next five years, through permanently leaving or reducing hours spent in direct patient care, or through taking a career break. GP age was an important predictor of career intentions; sharp increases in the proportion of GPs intending to quit patient care were evident from 52 years. Only 305 (14% weighted, 95%CI 13%, 16%) of GPs' reported high morale, while 54% (weighted, 95%CI 52%, 56%) reported low morale. Low morale was particularly common amongst GP partners. Current morale strongly predicted GPs' career intentions; those with very low morale were particularly likely to report intentions to quit patient care or to take a career break.

**Conclusions** A substantial majority of GPs in South West England report low morale. Many are considering career intentions which, if implemented, would adversely impact GP workforce capacity within a short time period.



For peer review only - http://bmjopen.bmj.com/site/about/guidelines.xhtml

**Study registration** NIHR HS&DR - 14/196/02, UKCRN ID 20700

## ARTICLE SUMMARY

Strengths and limitations of this study

- Census survey of all GPs in South West England, providing a cross-sectional overview of quit and career break intentions.
- High response rate, reflecting both rigorous planning and implementation of the survey, and interest among GPs in the subject matter of workforce challenges.
- The potential for non-response bias was reduced by weighting estimates to account for differential response rates by age, gender and practice role.
- The survey was cross-sectional, rather than longitudinal in design; previous work demonstrates that intention to quit is a strong predictor of actually leaving.
- The research was undertaken within a rapidly changing GP workforce policy and practice environment.

For peer review only



For peer review only - http://bmjopen.bmj.com/site/about/guidelines.xhtml

**INTRODUCTION**

UK healthcare has traditionally had a strong primary care base in which general practitioners (GPs) have a central role in providing care across many settings, including within general practices, in out-of-hours care, and in walk-in centres. Ninety percent of UK NHS patient contact takes place within primary care – 1.3m consultations every working day, 340m consultations per year, and with a projected primary care workload of 430m consultations per year by 2018(1, 2). Around 74% of primary care contacts take place with a general practitioner (GP). General practice has been described as "the jewel in the crown" of the NHS(3). GPs are trained, and have particular abilities in the diagnosis and management of patients with complex multi-morbidity.

UK general practice is, however, facing major problems regarding maintaining the GP workforce, with imminent GP shortages and a concomitant potential risk to patient care. A near quadrupling of unfilled GP posts was observed between 2010 and 2013 (from 2.1% to 7.9%), associated with an overall reduction in the number of GPs in England from 62 per 100,000 in 2009 to 59.5 in 2013(4). An estimated 12% of 2,947 GP training places were unfilled in England in 2013/14. These issues are compounded by an ageing GP workforce (30% of the 43,000 current GPs are over 50 years old(4)). Workforce issues are especially pertinent in inner city settings where recruitment and retention difficulties are further exacerbated by issues relating to the socio-demographic mix of the population and to increased demands for care.

Research commissioned by the BMA has highlighted the continuing recruitment and retention challenges. In a 2014 study of 431 doctors from the 2006 cohort of medical graduates, those in general practice reported the lowest morale, with higher than expected workload being identified as a key problem(5). Of male doctors, 90% plan to work full time compared with just 40% of female doctors(4). Younger GPs currently appear to be more reluctant to take on the financial risks and responsibilities of becoming a partner in a practice, leading to existing partners experiencing an increased workload(6). Recent surveys and reports have suggested that many GPs are considering permanently leaving direct patient care (1, 4, 7). Published evidence suggests that nearly half of GPs leaving general practice between 2009 and 2014 were under 50 years old(8).

There is thus an urgent need to find ways of retaining the GP workforce. If unaddressed, "meltdown" in NHS care may follow within the foreseeable future(1). The situation has been described as a "crisis"(9, 10) and there has been a call for policies and strategies to help retain GPs in direct patient care(4, 11). The future of NHS care over the next five years(12) is likely to involve new models of care, with innovations involving increasing professional skill mix and new approaches to managing



For peer review only - http://bmjopen.bmj.com/site/about/guidelines.xhtml

the service(12, 13) and in establishing federations of previously independent practices(12). It is vital that the GP workforce is sustained through this period of change.

Our research targets the critically important area of retention of experienced GPs in direct patient care, especially focussing on those GPs who may be considering retirement, and those GPs who have taken a career break (most often on account of family reasons). Following piloting of our questionnaire in 2015(14), this paper reports the findings of a recent census survey in which we aimed to describe GPs' career intentions, especially those which might impact on GP workforce availability over the next five years. The survey constitutes one part of a comprehensive programme of work, seeking to identify implementable policies and strategies to support the retention of experienced GPs in direct patient care and to support the return of GPs following a career break.

## METHODS

### Study sample

The sampling frame was the Medical Performers List of all GPs registered to practise in South West England, an area with a population of 3.5 million patients. National Performers Lists provide reassurance for the public that GPs, Dentists and Opticians practising in the NHS are suitably qualified, have up to date training, regulatory checks and appropriate English language skills(15). There were no exclusion criteria. The survey was administered between April and June 2016.

### Questionnaire

The questionnaire was based upon that used within earlier work (14), modified to increase the number of questions from 11 to 24, including rewording of four questions to reflect alignment of wording with other questionnaires of broadly similar intent, and by providing clear definitions for key concepts in the questionnaire including a career break, taking steps toward changing work-life balance and defining a clinical session. The questionnaire (Supplementary Online Material) comprised items that asked GPs about their career intentions, reporting on the likelihood that they would permanently leave direct patient care within the next two years, or within the next five years. GPs were also asked to report the likelihood that they would take a career break within the next five years, or that they would reduce their weekly average hours spent in direct patient care during this time period. GPs rated the likelihood of these events from “very likely” to “very unlikely” using a 4-point scale. The questionnaire also included a question about current level of morale, and captured general demographic data: gender, age, ethnicity, region and year of graduation, and current GP employment status (e.g., partner, salaried), number and pattern of sessions worked in a typical week, and involvement in delivering out-of-hours care.



For peer review only - http://bmjopen.bmj.com/site/about/guidelines.xhtml

**Data collection**

GPs were sent study materials through the post either to their practice or home address, and also by email, where available. The questionnaire was available for completion by post and online. The survey was supported by a comprehensive strategy of publicising the research through routine newsletters and circulars of relevant organisations and networks, including Local Medical Committees, Clinical Research Networks, Health Education England South West, the Royal College of General Practitioners, University of Exeter Medical School, and the South West Academic Health Science Network.

If a GP returned multiple online or postal surveys only the first response received by the research team was analysed. Postal response data were double entered and discrepancy checking was undertaken. Response data were stored securely and without participant name or address.

**Patient involvement**

Although the study participants were GPs rather than patients, patient representatives contributed to the design of the survey. The planned work was presented to the wider project's Patient and Public Involvement (PPI) group(16), by way of sharing the process and to check the integrity of the work, and the group provided supportive feedback. The survey results were presented at a project management group meeting, which included PPI representatives who directly contributed to interpreting and contextualising the results.

**Statistical analysis**

Differential response rates between different groups of GPs would potentially introduce bias into crude survey findings. To counter this we employed non-response weights. Inverse probability weights were calculated based on three factors: age (<40, 40-49, 50-54, 55-59, and 60 years and over), gender (male and female), and role (partner, salaried, and locum/other). By employing these weights we estimated what responses we would have received with a 100% response rate under the assumption that non-responders would have responded similarly to GPs of the same age, gender and role. Logistic regression was used to investigate the association between responses to questions regarding future career intentions (permanently leaving direct patient care within the next two and five years, taking a career break within the next five years, and reducing average hours spent in direct patient care within the next five years). Each of the four sets of responses was dichotomised into 'very likely' and 'likely' versus other responses. Initially, unadjusted associations were examined for effects attributable to the explanatory factors of gender, age, country of qualification, ethnicity, role/position, and rating of current morale. Subsequently, regression models adjusting simultaneously for all explanatory factors were used to examine adjusted associations. Similar



For peer review only - http://bmjopen.bmj.com/site/about/guidelines.xhtml

models were used with reported morale as the outcome (but not including morale as an explanatory factor). Regression analyses were restricted to those respondents with complete data on gender, age, country of qualification, ethnicity, role/position and rating of current morale.

**Supplementary analysis**

Interactions were explored between various factors in the models. Whilst some of these were found to be statistically significant, the magnitude of the interaction terms was generally small and did not alter the interpretation of the data, with one exception commented on in the results. For this reason we have not reported the more complex interaction models here.

In addition, we considered the possibility that some groups of GPs, for example female GPs, may not report the intention to reduce hours spent in direct patient care because they already work fewer hours, on average, than other groups. We therefore performed two supplementary analysis, including either a binary variable indicating part-time working (defined as working less than 8 sessions per week) or a continuous variable detailing the reported number of sessions worked per week.

All analyses were performed in Stata 14.2.

**RESULTS**

Questionnaires were distributed by post to 3370 GPs, with 1841 (55%) GPs also sent the questionnaire by email (Supplementary Online Material Table 1). Completed questionnaires were received from 2248/3370 GPs who were surveyed (response rate 67%). Of the 2248 GP respondents, 673 (30%) used the online survey. Response rates were as high for both males and females (67% and 66% respectively). Participation was lower amongst GPs aged under 40 years (54%) when compared with the older GPs aged 50 and over (in excess of 68% in each age group), and was lower for salaried GPs (57%) when compared with GP partners (71%) and non-principal/locum GPs (64%).

The median age of respondents was 48 years (Table 1; interquartile range (IQR) 40 to 55, range 28 to 84 years). Eighty-five percent of respondents reported having a practice with which they were primarily affiliated; 25% of respondents reported that they were involved in the delivery of out-of-hours primary medical care. The majority (62%) of respondents were partners in their practices.



For peer review only - http://bmjopen.bmj.com/site/about/guidelines.xhtml

**Table 1 Characteristics of responding GPs (N=2248)**

| | | Proportion; n (%) |
|---|---|---|
| Gender | Male | 1053 (46.8) |
| | Female | 1190 (52.9) |
| | Prefer not to say | 3 (0.1) |
| | Missing | 2 (0.1) |
| Age (years) | Under 40 | 497 (22.1) |
| | 40-49 | 735 (32.7) |
| | 50-54 | 394 (17.5) |
| | 55–59 | 408 (18.2) |
| | 60 or over | 209 (9.3) |
| | Missing | 4 (0.2) |
| | Spoiled | 1 (0.0) |
| Ethnic group | White | 2100 (93.4) |
| | Mixed/multiple ethnic groups | 29 (1.3) |
| | Asian/Asian British | 78 (3.5) |
| | Black/African/Caribbean/Black British | 9 (0.4) |
| | Other ethnic group | 19 (0.9) |
| | Missing | 13 (0.6) |
| Region of qualification | UK/Ireland | 2107 (93.7) |
| | Europe (non-UK/Ireland) | 70 (3.1) |
| | South Asia | 21 (0.9) |
| | Other | 42 (1.9) |
| | Missing | 7 (0.3) |
| | Spoiled | 1 (0.0) |
| Role/position | GP Partner | 1403 (62.4) |
| | Salaried GP | 454 (20.2) |
| | Locum GP | 287 (12.8) |
| | Other | 68 (3.0) |
| | Missing | 7 (0.3) |
| | Spoiled | 29 (1.3) |

**Career intentions**

Of the 2248 responding GPs, 55 had already permanently left direct patient care (Table 2); 16 had selected 'none of the above' to reflect their working status in direct patient care – i.e. that they were not currently working in direct patient care, nor on a career break, but had not permanently quit – and were removed from further analysis. Differential response by age and role meant that GP groups reporting an intention to quit direct patient care were somewhat overrepresented, introducing a small bias into the crude results which is accounted for in the weighted percentages.

Of the 2179 GP participants included in the analysis, 473 (weighted percentage 20.3%, 95%CI 18.7%, 22.0%) reported a high likelihood of quitting direct patient care ('likely' or 'very likely') within the next two years, and 863 (weighted percentage 36.7%, 95%CI 34.7%, 38.8%) within the next five years (Table 2). There were 1252 (weighted percentage 56.7%, 95%CI 54.5%, 58.8%) participants who reported being likely or very likely to reduce hours, and 770 (weighted percentage 36.2%, 95%CI 61.6%, 65.8%) who reported the intention to take a career break within the next five years.



For peer review only - http://bmjopen.bmj.com/site/about/guidelines.xhtml

Considered together, 1535 (weighted percentage 69.9%, 95%CI 67.9%, 71.8%) participants reported that they were likely/very likely to pursue a career intention (one or more of the four presented) that would potentially adversely impact the workforce available in South West England within the next five years. The majority of participants also had low morale, with a substantially greater proportion of participants (1195 - weighted percentage 54.4%, 95%CI 52.2%, 56.5%) reporting 'low' or 'very low' levels of morale compared with only 305 (weighted percentage 14.2%, 95%CI 12.7%, 15.7%) reporting 'high' or 'very high' morale.

**Associations between GP characteristics and career intentions/morale**

Figure 1 illustrates the reported intention to quit direct patient care of responding GPs broken down by gender and each year of GP age, aggregating likely and very likely responses together. Reported quit intentions are strongly related to age, remaining low (<20%) among younger GPs (45 years and under) for quitting direct patient care in both of the next two and five years. Both of these outcomes show a sharp rise from the age of 52 years, with the proportion stating they were likely to quit in five years rising to almost 90% by the age of 56 years. The proportion anticipating taking a career break as 'likely' was highest for the youngest GPs, especially amongst younger women where nearly 9 out of 10 female GPs aged 30 years reported the intention to take a career break, presumably on account of anticipated maternity leave.

Of the 2179 respondents reporting that their current role involved direct patient care, 2119 (97%) provided complete information on gender, age, region of qualification, ethnicity, role/position and rating of current morale and were included in the regression analyses. Tables 3 and 4 show the results of the unadjusted and adjusted logistic regression analyses respectively, for career intentions. In unadjusted analyses there is strong evidence (p<0.001 for all) that gender and role are associated with being likely to report the intention to leave patient care within both two and five years, with female GPs being less likely to quit patient care than male GPs (OR 0.55 95% CI 0.46, 0.66 for leaving patient care in five years), and with locum GPs being the most likely to quit patient care and salaried GPs least likely. However, these associations are likely be confounded, for example by the fact younger doctors were more likely to be female compared with older doctors. Once adjustment is made for all other factors, the effects of these characteristics on intentions to quit are no longer significant. However, after adjustment for other variables, women were still substantially less likely to report intending to reduce hours spent in direct patient care (OR 0.58 95% CI 0.47, 0.71), and locum GPs remained more likely to report intending to take a career break when compared with GPs in other roles.



For peer review only - http://bmjopen.bmj.com/site/about/guidelines.xhtml

**Table 2 Career intentions of responding GPs (n=2179)**

| | How likely is it that you will permanently leave direct patient care within the next 2 years? | | | How likely is it that you will permanently leave direct patient care within the next 5 years? | | | How likely is it that you will reduce your weekly average hours spent in direct patient care within the next 5 years? | | | How likely is it that you will take a career break (or another career break) within the next 5 years? | | |
|---|---|---|---|---|---|---|---|---|---|---|---|---|
| | n | % crude | % Weighted (95% CI) | n | % crude | % weighted (95% CI) | n | % crude | % weighted (95% CI) | n | % crude | % weighted (95% CI) |
| Very likely | 255 | 11.7 | 10.8 (9.6, 12.1) | 607 | 27.9 | 25.4 (23.6, 27.2) | 773 | 35.5 | 34.0 (32.0, 36.0) | 453 | 20.8 | 20.7 (19.0, 22.5) |
| Likely | 218 | 10.0 | 9.5 (8.4, 10.8) | 256 | 11.7 | 11.4 (10.1, 12.8) | 479 | 22.0 | 22.7 (20.9, 24.5) | 317 | 14.5 | 15.6 (14.0, 17.2) |
| Unlikely | 795 | 36.5 | 36.6 (34.6, 38.7) | 675 | 31.0 | 32.3 (30.3, 34.3) | 585 | 26.8 | 27.5 (25.7, 29.5) | 756 | 34.7 | 34.9 (32.9, 36.9) |
| Very unlikely | 899 | 41.3 | 42.6 (40.5, 44.7) | 622 | 28.5 | 30.1 (28.2, 32.1) | 326 | 15.0 | 15.2 (13.7, 16.8) | 614 | 28.2 | 27.3 (25.4, 29.2) |
| Missing | 12 | 0.6 | 0.5 (0.3, 1.0) | 17 | 0.8 | 0.8 (0.0, 0.4) | 15 | 0.7 | 0.6 (0.0, 0.2) | 36 | 1.7 | 1.5 (1.1, 2.1) |
| Spoiled | 0 | 0.0 | 0.0 | 2 | 0.1 | 0.1 (0.5, 1.2) | 1 | 0.0 | 0.0 (0.4, 1.0) | 3 | 0.1 | 0.1 (0.0, 0.4) |

| | How would you describe your current level of morale? | | |
|---|---|---|---|
| Very low | 352 | 16.2 | 15.6 (14.1, 17.2) |
| Low | 843 | 38.7 | 38.8 (36.7, 40.9) |
| Neither low nor high | 664 | 30.5 | 30.8 (28.8, 32.8) |
| High | 276 | 12.7 | 12.9 (11.6, 14.4) |
| Very high | 29 | 1.3 | 1.3 (0.9, 1.8) |
| Missing | 11 | 0.5 | 0.5 (0.1, 0.5) |
| Spoiled | 4 | 0.2 | 0.2 (0.3, 0.9) |

For peer review only

Age was a very strong predictor of reported intention to leave direct patient care, a finding which persisted after adjustment (for example 60-69 vs. 40-49 year olds OR=194.4 95% CI 84.3, 448.3 for leaving patient care in five years). Age was also a strong predictor of the intention to reduce hours in the adjusted model, with older GPs more likely to report this intention than younger GPs. Female GPs, GPs aged under 40 years, and locum GPs were most likely to report the intention to take a career break after adjustment for other factors. A model including an interaction between age and gender showed that the effect of under 40 year olds being more likely to report intentions to take a career break was strongest in female GPs, consistent with the pattern shown in Figure 1 (results not shown).

Self-reported morale was a strong predictor of all four outcomes shown in Tables 2 and 3. In the unadjusted analysis a 'U-shaped' relationship is seen for all four quitting outcomes, with those with 'very high' morale more likely to report intentions to leave direct patient care/reduce hours/take a career break than those with 'high' or 'neither low nor high' morale (and in the case of leaving patient care in two years, more likely than any other morale category), but less likely to intend to leave/reduce hours/take a career break than those with 'very low' morale. This U-shaped relationship largely disappears however after adjustment, and it is those GPs with 'very low' morale who are most likely to report intending to leave direct patient care, reduce hours in patient care, or to take a career break. Moreover, the change in odds between 'low' and 'very low' morale is particularly strong. Table 5 shows the results of the logistic regression analysis modelling of factors associated with low or very low morale. Only age and practice role show any evidence of being associated with reported morale ($p<0.001$ for both). Those aged 50-54 years old are most likely to report low morale. In respect of role, GP partners are the most likely to report low morale, followed by salaried GPs, and with locums and other GPs the least likely groups to report low morale.

Supplementary analyses exploring the contribution of current working patterns to any association with intentions to reduce hours spent in direct patient care did explain some of the difference between genders. However, even after adjustment for current working patterns, female GPs were still substantially less likely to report intentions to reduce hours spent in direct patient care than their male counterparts (not shown).



For peer review only - http://bmjopen.bmj.com/site/about/guidelines.xhtml

**Table 3 Unadjusted associations between career intentions and GP attributes**

| | | How likely is it that you will permanently leave direct patient care within the next 2 years? | | How likely is it that you will permanently leave direct patient care within the next 5 years? | | How likely is it that you will reduce your weekly average hours spent in direct patient care within the next 5 years? | | How likely is it that you will take a career break (or another career break) within the next 5 years? | |
|---|---|---|---|---|---|---|---|---|---|
| | | OR† (95% CI) | p | OR† (95% CI) | p | OR† (95% CI) | p | OR† (95% CI) | p |
| Gender | Male | Reference | <0.001 | Reference | <0.001 | Reference | <0.001 | Reference | 0.046 |
| | Female | 0.51 (0.41, 0.63) | | 0.55 (0.46, 0.66) | | 0.48 (0.41, 0.58) | | 1.20 (1.00, 1.44) | |
| Age | <40 | 0.75 (0.48, 1.16) | <0.001 | 0.49 (0.34, 0.70) | <0.001 | 1.24 (0.98, 1.56) | <0.001 | 2.34 (1.83, 2.98) | <0.001 |
| | 40-49 | Reference | | Reference | | Reference | | Reference | |
| | 50-54 | 1.65 (1.12, 2.43) | | 4.00 (3.02, 5.28) | | 2.04 (1.58, 2.63) | | 1.24 (0.95, 1.63) | |
| | 55-59 | 9.32 (6.72, 12.92) | | 30.04 (21.04, 42.90) | | 5.00 (3.74, 6.69) | | 1.59 (1.22, 2.08) | |
| | 60-69 | 22.22 (14.43, 34.22) | | 91.60 (41.88, 200.36) | | 7.38 (4.58, 11.88) | | 1.61 (1.11, 2.35) | |
| | 70+ | 45.86 (12.84, 163.84) | | * | | 3.86 (1.23, 12.10) | | 1.55 (0.56, 4.32) | |
| Country of qualification | UK/Ireland | Reference | 0.450 | Reference | 0.132 | Reference | 0.921 | Reference | 0.470 |
| | Europe | 0.56 (0.27, 1.14) | | 0.60 (0.35, 1.02) | | 0.85 (0.52, 1.39) | | 0.65 (0.37, 1.12) | |
| | South Asia | 0.88 (0.29, 2.66) | | 0.43 (0.14, 1.32) | | 1.10 (0.45, 2.70) | | 0.95 (0.38, 2.39) | |
| | Other | 1.05 (0.47, 2.32) | | 0.98 (0.51, 1.90) | | 0.95 (0.50, 1.80) | | 0.88 (0.45, 1.73) | |
| Ethnic group | White | Reference | 0.300 | Reference | 0.032 | Reference | 0.581 | Reference | 0.610 |
| | Mixed | * | | 0.60 (0.26, 1.36) | | 0.74 (0.35, 1.57) | | 1.57 (0.74, 3.31) | |
| | Asian | 0.87 (0.48, 1.57) | | 0.43 (0.25, 0.76) | | 1.27 (0.78, 2.05) | | 1.00 (0.61, 1.63) | |
| | Black | 3.59 (0.89, 14.41) | | 1.49 (0.37, 5.99) | | 2.23 (0.45, 11.07) | | 0.60 (0.12, 2.99) | |
| | Other | 0.77 (0.22, 2.69) | | 0.68 (0.24, 1.96) | | 1.36 (0.50, 3.70) | | 1.61 (0.62, 4.18) | |
| Role/ Position | GP Partner | Reference | <0.001 | Reference | <0.001 | Reference | <0.001 | Reference | <0.001 |
| | Salaried GP | 0.61 (0.46, 0.83) | | 0.43 (0.34, 0.55) | | 0.63 (0.51, 0.78) | | 1.25 (1.00, 1.56) | |
| | Locum GP | 1.58 (1.18, 2.11) | | 1.08 (0.82, 1.41) | | 1.34 (1.02, 1.76) | | 1.72 (1.32, 2.24) | |
| | Other | 1.12 (0.61, 2.08) | | 0.72 (0.40, 1.26) | | 0.79 (0.47, 1.34) | | 0.85 (0.48, 1.52) | |
| Reported morale | Very low | 0.99 (0.45, 2.17) | <0.001 | 1.85 (0.81, 4.19) | <0.001 | 2.58 (1.19, 5.59) | <0.001 | 2.24 (1.01, 4.95) | <0.001 |
| | Low | 0.42 (0.19, 0.91) | | 0.78 (0.35, 1.75) | | 1.31 (0.62, 2.77) | | 1.14 (0.52, 2.49) | |
| | Neither low/high | 0.30 (0.14, 0.65) | | 0.62 (0.28, 1.40) | | 0.82 (0.39, 1.73) | | 0.81 (0.37, 1.78) | |
| | High | 0.40 (0.18, 0.90) | | 0.62 (0.27, 1.42) | | 0.54 (0.25, 1.16) | | 0.55 (0.24, 1.24) | |
| | Very high | Reference | | Reference | | Reference | | Reference | |

† ORs>1 indicate category is more likely to leave direct patient care/reduce hours/take a career break than the reference category
* This group has been excluded from the analysis due to perfect prediction, i.e. all over 70 year olds reported being likely to leave direct patient in 5 years and no mixed ethnicity GPs reported being likely to leave direct patient care in 2 years



For peer review only - http://bmjopen.bmj.com/site/about/guidelines.xhtml

For peer review only

**Table 4 Adjusted associations between career intentions and GP attributes. Adjustment was made for all factors shown in table.**

| | | How likely is it that you will permanently leave direct patient care within the next 2 years? | | How likely is it that you will permanently leave direct patient care within the next 5 years? | | How likely is it that you will reduce your weekly average hours spent in direct patient care within the next 5 years? | | How likely is it that you will take a career break (or another career break) within the next 5 years? | |
|---|---|---|---|---|---|---|---|---|---|
| | | OR† (95% CI) | p | OR† (95% CI) | p | OR† (95% CI) | p | OR† (95% CI) | p |
| Gender | Male | Reference | 0.181 | Reference | 0.810 | Reference | <0.001 | Reference | 0.019 |
| | Female | 0.83 (0.64, 1.09) | | 1.03 (0.80, 1.33) | | 0.58 (0.47, 0.71) | | 1.27 (1.04, 1.55) | |
| Age | <40 | 0.89 (0.56, 1.41) | <0.001 | 0.58 (0.40, 0.85) | <0.001 | 1.47 (1.14, 1.90) | <0.001 | 2.63 (2.02, 3.42) | <0.001 |
| | 40-49 | Reference | | Reference | | Reference | | Reference | |
| | 50-54 | 1.70 (1.14, 2.55) | | 4.73 (3.49, 6.41) | | 2.11 (1.61, 2.77) | | 1.31 (0.98, 1.73) | |
| | 55-59 | 11.69 (8.18, 16.72) | | 44.15 (29.77, 65.48) | | 5.62 (4.12, 7.66) | | 1.79 (1.35, 2.38) | |
| | 60-69 | 36.87 (22.53, 60.34) | | 194.43 (84.32, 448.34) | | 9.58 (5.76, 15.95) | | 2.03 (1.35, 3.04) | |
| | 70+ | 110.61 (25.85, 473.26) | | * | | 4.93 (1.47, 16.55) | | 1.83 (0.61, 5.48) | |
| Country of qualification | UK/Ireland | Reference | 0.193 | Reference | 0.048 | Reference | 0.829 | Reference | 0.738 |
| | Europe | 0.60 (0.27, 1.37) | | 0.48 (0.23, 0.99) | | 0.98 (0.57, 1.70) | | 0.73 (0.41, 1.29) | |
| | South Asia | 0.21 (0.04, 1.15) | | 0.13 (0.02, 0.97) | | 0.61 (0.19, 1.96) | | 0.98 (0.31, 3.05) | |
| | Other | 0.83 (0.30, 2.31) | | 0.94 (0.39, 2.25) | | 0.83 (0.40, 1.71) | | 1.08 (0.53, 2.22) | |
| Ethnic group | White | Reference | 0.109 | Reference | 0.142 | Reference | 0.209 | Reference | 0.483 |
| | Mixed | * | | 2.84 (1.15, 7.04) | | 1.32 (0.60, 2.92) | | 1.74 (0.79, 3.81) | |
| | Asian | 1.58 (0.70, 3.54) | | 0.84 (0.38, 1.86) | | 1.89 (1.04, 3.46) | | 0.87 (0.48, 1.57) | |
| | Black | 6.07 (1.00, 36.99) | | 2.26 (0.29, 17.71) | | 2.89 (0.48, 17.28) | | 0.54 (0.10, 2.87) | |
| | Other | 0.45 (0.10, 2.16) | | 0.45 (0.08, 2.43) | | 1.17 (0.39, 3.45) | | 1.57 (0.56, 4.41) | |
| Role/ Position | GP Partner | Reference | 0.177 | Reference | 0.153 | Reference | 0.005 | Reference | <0.001 |
| | Salaried GP | 1.10 (0.76, 1.59) | | 0.81 (0.58, 1.14) | | 0.97 (0.75, 1.24) | | 1.15 (0.89, 1.47) | |
| | Locum GP | 1.55 (1.04, 2.30) | | 1.37 (0.91, 2.07) | | 1.74 (1.26, 2.39) | | 2.09 (1.55, 2.81) | |
| | Other | 0.91 (0.39, 2.15) | | 0.73 (0.29, 1.86) | | 1.19 (0.64, 2.20) | | 1.15 (0.62, 2.13) | |
| Reported morale | Very low | 5.77 (1.96, 17.00) | <0.001 | 13.07 (3.72, 45.88) | <0.001 | 6.74 (2.76, 16.47) | <0.001 | 3.49 (1.49, 8.14) | <0.001 |
| | Low | 1.87 (0.65, 5.38) | | 3.89 (1.13, 13.34) | | 3.27 (1.38, 7.74) | | 1.50 (0.66, 3.45) | |
| | Neither low/high | 0.86 (0.30, 2.47) | | 2.15 (0.63, 7.39) | | 1.75 (0.74, 4.14) | | 0.93 (0.40, 2.12) | |
| | High | 0.80 (0.27, 2.39) | | 1.29 (0.36, 4.60) | | 0.86 (0.36, 2.08) | | 0.59 (0.25, 1.40) | |
| | Very high | Reference | | Reference | | Reference | | Reference | |

† ORs>1 indicate category is more likely to leave direct patient care/reduce hours/take a career break than the reference category

* This group has been excluded from the analysis due to perfect prediction, i.e. all over 70 year olds reported being likely to leave direct patient in 5 years and no mixed ethnicity GPs reported being likely to leave direct patient care in 2 years



For peer review only

For peer review only - http://bmjopen.bmj.com/site/about/guidelines.xhtml

**Table 5 Unadjusted and adjusted associations between self-reported low or very low morale and GP attributes. Adjustment was made for all factors shown in table.**

| | | Unadjusted | | Adjusted | |
|---|---|---|---|---|---|
| | | OR† (95% CI) | p | OR† (95% CI) | p |
| Gender | Male | Reference | 0.134 | Reference | 0.231 |
| | Female | 0.87 (0.72 ,0.72) | | 0.90 (0.76 ,0.76) | |
| Age | <40 | 0.73 (0.57 ,0.57) | <0.001 | 0.69 (0.55 ,0.55) | <0.001 |
| | 40-49 | Reference | | Reference | |
| | 50-54 | 1.06 (0.81 ,0.81) | | 1.10 (0.85 ,0.85) | |
| | 55-59 | 0.85 (0.66 ,0.66) | | 0.92 (0.71 ,0.71) | |
| | 60-69 | 0.38 (0.26 ,0.26) | | 0.33 (0.23 ,0.23) | |
| | 70+ | 0.07 (0.01 ,0.01) | | 0.04 (0.01 ,0.01) | |
| Country of qualification | UK/Ireland | Reference | 0.157 | Reference | 0.194 |
| | Europe | 0.74 (0.45 ,0.45) | | 0.73 (0.45 ,0.45) | |
| | South Asia | 0.97 (0.32 ,0.32) | | 0.97 (0.40 ,0.40) | |
| | Other | 0.50 (0.25 ,0.25) | | 0.55 (0.29 ,0.29) | |
| Ethnic group | White | Reference | 0.534 | Reference | 0.721 |
| | Mixed | 0.82 (0.38 ,0.38) | | 0.82 (0.39 ,0.39) | |
| | Asian | 1.59 (0.89 ,0.89) | | 1.31 (0.81 ,0.81) | |
| | Black | 1.64 (0.37 ,0.37) | | 0.82 (0.20 ,0.20) | |
| | Other | 1.01 (0.36 ,0.36) | | 0.73 (0.28 ,0.28) | |
| Role/ Position | GP Partner | Reference | <0.001 | Reference | <0.001 |
| | Salaried GP | 0.75 (0.59 ,0.59) | | 0.67 (0.54 ,0.54) | |
| | Locum GP | 0.40 (0.30 ,0.30) | | 0.33 (0.25 ,0.25) | |
| | Other | 0.22 (0.12 ,0.12) | | 0.20 (0.11 ,0.11) | |

† ORs>1 indicate category is more likely to report low or very low morale than the reference category

For peer review only

## DISCUSSION

### Main findings

A high proportion of GPs – around two in every five – currently working within direct patient care in South West England reported an intention to permanently quit direct patient care within the next five years, this being one in five within the next two years. Further depletion of the GP workforce in this region of England through reduction of weekly average hours or through taking a career break within the next five years also emerged as an impending risk. Overall, seven out of every ten GPs in this region reported a career intention which, if implemented, would adversely impact the GP workforce capacity in South West England through GPs leaving direct patient care, reducing hours spent in direct patient care, or by taking a career break within the next five years.

Older age is highly predictive of a GP's intentions to permanently quit direct patient care and to reduce hours. The intention to quit was independent of the GP's role (partner, salaried or locum) and gender. The gender differences observed amongst younger GPs in respect of intended career breaks is a particular issue where, as currently exists, 69% of the GP trainee workforce is female(17), and where a substantial proportion of younger women returning to clinical care do so on the basis of reduced hours(1), and where doctors who wish to return to clinical practice may face significant bureaucratic obstacles in doing so(18). The decision to reduce hours or to take a career break varied with employment status, with locum GPs being most likely to report intentions to reduce hours or to take a career break. In a situation where GPs who are not partners in a practice do in fact leave direct patient care, the system of care appears vulnerable, with added strain likely to be placed on GPs who are partners in their practice.

A key issue is whether the intention to leave is translated into reality, and few studies have explored this important question. However, Hann et al (19) explored this question amongst nearly 1200 UK GPs followed up over five years, and identified a strong relationship between a stated intention to leave patient care and actually doing so, reflected in a 4.5-fold difference in odds of leaving between those with no stated intention to leave patient care when compared with those reporting a high intention to do so.

The majority of responding GPs in our study reported low morale, with less than 15% reporting high morale. GP morale was identified as an important predictor of future career intentions, particularly when morale was very low.



For peer review only - http://bmjopen.bmj.com/site/about/guidelines.xhtml

**Strengths and limitations of the study**

By ensuring alignment of wording with other surveys of similar intent allows the opportunity for comparing and contrasting of results. Recent high quality surveys support our estimate of the proportion of GPs intending to leave general practice within the next five years (14, 20-22). Unlike most other similar surveys which tend to survey samples of GPs(7, 23), we undertook a census survey of all GPs currently eligible to provide patient care across a large area, providing a cross-sectional overview of the quit intentions of all GPs in South West England. The response rate was high (67%), reflecting both rigorous planning and implementation of the survey, and interest among GPs in the subject matter of workforce challenges. Further, we have reduced the potential for non-response bias by weighting our estimates to account for differential response rates by age, gender and practice role. The overall programme of work, of which this survey is one element, includes the planned development of a predictive risk modelling tool to enumerate risk of future workforce undersupply at local levels, thus offering the potential of providing data that will be of value to healthcare planners and policy makers. In addition, our study relates to a key current area of national interest and concern.

The study has a number of limitations. The survey was conducted amongst GPs in a single region of England which may not be entirely typical of England as a whole, for example in respect of population-mix and practice setting(24). The region is often informally regarded as having desirable living and working conditions(25). It seems likely that other regions of England, with more inner-city areas with higher levels of deprivation, are likely to face even greater challenges in respect of the GP workforce than in the findings we have presented here. A further limitation is that the research was undertaken within a rapidly changing GP workforce policy environment, for example, the implementation in 2015-16 of a 10-point plan agreed between authoritative UK health, governmental, and professional bodies concerned about GP workforce issues(26). Finally, this survey was cross-sectional, rather than longitudinal in design, and so we are unable to report on the actual translation of GPs' reported career intentions into actual career decisions, although previous work has demonstrated intention to quit to be a strong predictor of actually leaving(19).

**Implications for research**

Our survey is part of a multi-method suite of work commissioned by the NHS, seeking to inform evidence-based policies and strategies in a key area of national concern, and which will involve stakeholder consultations with policy-makers who have oversight of GP workforce planning(16). Future research should include assessment of the predictive validity of GPs' reported career intentions by longitudinal follow-up of GPs, investigation of how reducing hours or better flexible



For peer review only - http://bmjopen.bmj.com/site/about/guidelines.xhtml

working arrangements may influence GP retention, and undertaking research exploring the potential for extended clinical roles amongst important allied healthcare professionals in the clinical workforce. Such groups might include nurses, physiotherapists and pharmacists, and developing such extended roles for these professionals within the primary care team may offer a rapid and potentially effective contribution to the alleviation of current GP workforce pressures. Research into the determinants and impact of working reduced hours should also encompass other aspects of flexible working arrangements in general practice, and how such arrangements may improve job satisfaction and work-life balance, and thereby potentially increase GP retention in the longer term. This work also provides insight into which groups of GPs might best be targeted with interventions aiming to improve workforce retention.

**Implications for policy and practice**

Concerns regarding the GP workforce are now recognised widely by authoritative bodies in the UK, leading to recent policy statements and initiatives(26) and to the establishment of a Primary Care Workforce Commission by Health Education England(4). Our research can inform this policy agenda by providing an estimate of the likely proportion of South West GPs intending to leave direct patient care within the next five years through permanently quitting patient care or by taking a career break. Our findings are likely to be of relevance in other areas of England that may face even greater challenges in respect of the GP workforce. Retaining the direct patient care capacity of established GPs represents an immediate challenge for healthcare planners. Recognising and addressing issues of low GP morale is an area of importance, and addressing the issue may not only improve how GPs feel about their work, but also potentially be associated with improvements in patients' experience of the care received(27). In the face of problems recruiting to general practice(28) and the wider workforce problems, the UK government recently announced an increase in the number of training places for GPs(26) and an increase in the number of medical training places in the UK(29). But these medium- to long-term solutions do not address the immediate workforce crisis. In contrast, policy initiatives to support the retention of the existing GP workforce, the rapid development of and support for new models of care, and the implementation of policies aimed at alleviating workload pressures for GPs, are potential short- to medium-term solutions which need to be urgently considered. Developing and implementing relevant policies and strategies to achieve those ambitions will be important for the alleviation of the immediate pressures faced by the GP workforce and the populations of patients they serve.

**Conclusions**



For peer review only - http://bmjopen.bmj.com/site/about/guidelines.xhtml

Healthcare in England faces imminent challenges in respect of GP workforce capacity. This survey identifies the magnitude of the problem in South West England and highlights the important role of GP morale as one important factor contributing to that challenge. Acknowledgement of the magnitude of the problems is urgently required, along with implementation and monitoring of relevant policy and strategy.  Failure to do so will risk serious adverse effects on the capacity and ability of the NHS to provide effective primary care to the UK population.

For peer review only



For peer review only - http://bmjopen.bmj.com/site/about/guidelines.xhtml

**Acknowledgements**

We are grateful to Dr Nigel Acheson and Dr Liz Thomas (NHS England) for their support with the conduct of this research, to all of the GP participants who contributed survey responses, to the PPI group chaired by Dr Jo Welsman who support the wider programme of work of which the survey is part, to Alex Aylward, patient representative, who commented on survey design, and to Dr Jo Roberts, (South West Academic Health Science Network and South Devon CCG) and other members of the study advisory group.

**Contributors**

EF contributed to the study design, data collection, analysis and writing of the paper. JC contributed to study design, analysis and writing of the paper. RA, SR, CS, SD, AS and FW contributed to the study design and writing of the paper. GA contributed to analysis and writing of the paper. All authors read and approved the final paper. JC is the guarantor of the paper.

**Funding**

The project was funded by the National Institute for Health Research, Health Service and Delivery Research programme (project 14/196/02). The views and opinions expressed herein are those of the authors and do not necessarily reflect those of the Health Service and Delivery Research programme, the National Institute for Health Research, the National Health Service, or the Department of Health.

**Ethical approval**

This study was approved by the University of Exeter Medical School Research Ethics Committee (15/11/085).

**Competing interests**

All authors have completed the ICMJE uniform disclosure form at www.icmje.org/coi_disclosure.pdf (available on request from the corresponding author) and declare: grants from the National Institute for Health Research (Health Service and Delivery Research programme) during the conduct of the study. SD's position is partly supported by the National Institute for Health Research (NIHR) Collaboration for Leadership in Applied Health Research and Care South West Peninsula at the Royal Devon and Exeter NHS Foundation Trust. The views expressed are those of the author(s) and not necessarily those of the NHS, the NIHR or the Department of Health.



For peer review only

For peer review only - http://bmjopen.bmj.com/site/about/guidelines.xhtml

**Data sharing**

Requests for statistical code and dataset can be made to the corresponding author at john.campbell@exeter.ac.uk. The dataset will be made available via a publicly accessible repository on publication.

All authors, external and internal, had full access to all of the data (including statistical reports and tables) in the study and can take responsibility for the integrity of the data and the accuracy of the data analysis.

**Transparency**

The lead author (EF) and corresponding author (JC) affirm that the manuscript is an honest, accurate, and transparent account of the study being reported, and that no aspects of the study have been omitted.

The Corresponding Author has the right to grant on behalf of all authors and does grant on behalf of all authors, a worldwide licence (http://www.bmj.com/sites/default/files/BMJ%20Author%20Licence%20March%202013.doc) to the Publishers and its licensees in perpetuity, in all forms, formats and media (whether known now or created in the future), to i) publish, reproduce, distribute, display and store the Contribution, ii) translate the Contribution into other languages, create adaptations, reprints, include within collections and create summaries, extracts and/or, abstracts of the Contribution and convert or allow conversion into any format including without limitation audio, iii) create any other derivative work(s) based in whole or part on the on the Contribution, iv) to exploit all subsidiary rights to exploit all subsidiary rights that currently exist or as may exist in the future in the Contribution, v) the inclusion of electronic links from the Contribution to third party material where-ever it may be located; and, vi) licence any third party to do any or all of the above. All research articles will be made available on an Open Access basis (with authors being asked to pay an open access fee—see http://www.bmj.com/about-bmj/resources-authors/forms-policies-and-checklists/copyright-open-access-and-permission-reuse). The terms of such Open Access shall be governed by a Creative Commons licence—details as to which Creative Commons licence will apply to the research article are set out in our worldwide licence referred to above.

**References**

1. Centre for Workforce Intelligence. In-depth review of the general practitioner workforce: final report. London; 2014.
2. Hobbs FR, Bankhead C, Mukhtar T, Stevens S, Perera-Salazar R, Holt T, et al. Clinical workload in UK primary care: a retrospective analysis of 100 million consultations in England, 2007–14. The Lancet. 2016;387(10035):2323-30.



For peer review only - http://bmjopen.bmj.com/site/about/guidelines.xhtml

3. Berwick DM. A transatlantic review of the NHS at 60. British Medical Journal,. 2008;337(7663):212-4.
4. Health Education England (HEE) Commission on GP workforce. Securing the Future GP Workforce - Delivering the Mandate on GP Expansion. London: Health Education England; 2014.
5. British Medical Association. BMA cohort study of 2006 medical graduates. Eighth report. London; 2014.
6. Sansom A, Calitri R, Carter M, Campbell J. Understanding quit decisions in primary care: a qualitative study of older GPs. BMJ Open. 2016;6(2).
7. Hann M, McDonald J, Checkland K, Coleman A, Gravelle H, Sibbald B, et al. Seventh national GP worklife survey. Manchester: University of Manchester; 2013.
8. NHS Digital. General and personal medical services, England – 2004–2014 as at 30 September Leeds: NHS Digital; 2015 [Available from: http://content.digital.nhs.uk/catalogue/PUB16934.
9. Dayan M, Arora S, Rosen R, Curry N. Is general practice in crisis? London: Nuffield Trust; 2014.
10. Baird B, Charles,A., Honeyman, M., Maguire, D., Das, P. Understanding pressures in general practice London: King's Fund; 2016 [updated 1/12/2016. Available from: https://www.kingsfund.org.uk/sites/files/kf/field/field_publication_file/Understanding-GP-pressures-Kings-Fund-May-2016.pdf.
11. Pulse. RCGP petitions Government to create new 'medical assistant' role to relieve admin pressures on GPs 2014 [Available from: http://www.pulsetoday.co.uk/20008118.article#.VD_Iv01wbcs.
12. NHS. Five year forward view. London: NHS England; 2014.
13. The King's Fund and Nuffield Trust. Securing the Future of General Practice: New Models of Primary Care London2013 [Available from: http://www.nuffieldtrust.org.uk/publications/securing-future-general-practice.
14. Campbell J, Calitri, R., Sansom, A. Retaining the experienced GP workforce in direct patient care (ReGROUP): Final Report. Exeter: University of Exeter; 2015.
15. NHS England National Performers List London2016 [Available from: https://www.performer.england.nhs.uk/.
16. Primary Care Research Group UoEMS. The changing general practitioner worforce: the development of policies and strategies aimed at retaining experienced GPs and those taking a career break in direct patient care: ReGROUP project 2016 [Available from: http://medicine.exeter.ac.uk/research/healthresearch/regroup/.
17. General Medical Council. The state of medical education and practice in the UK. London; 2015.
18. Royal College of General Practitioners. Thousands of doctors prevented from returning to GP workforce by red tape London: RCGP; 2014 [Available from: http://www.rcgp.org.uk/news/2014/august/thousands-of-doctors-prevented-from-returning-to-gp-workforce-by-red-tape.aspx.
19. Hann M, Reeves D, Sibbald B. Relationships between job satisfaction, intentions to leave family practice and actually leaving among family physicians in England. European journal of public health. 2011;21(4):499-503.
20. Gibson J, Checkland K, Coleman A, Hann M, McCall R, Spooner S, et al. Eighth national GP worklife survey. Manchester; 2015.
21. Dale J, Potter R, Owen K, Parsons N, Realpe A, Leach J. Retaining the general practitioner workforce in England: what matters to GPs? A cross-sectional study. BMC family practice. 2015;16.
22. Martin SD, E. Gershlick, B. Under pressure: What the Commonwealth Fund's 2015 international survey of general practitioners means for the UK. London: The Health Foundation; 2016.
23. British Medical Association. BMA quarterly tracker survey: current views from across the medical profession. Quarter 3: July/August 2014. London; 2014.



For peer review only - http://bmjopen.bmj.com/site/about/guidelines.xhtml

24. UK Government. English Indices of Deprivation 2015 2015 [Available from: https://www.gov.uk/government/statistics/english-indices-of-deprivation-2015.

25. wikipedia. South West England 2016 [Available from: https://en.wikipedia.org/wiki/South_West_England#Deprivation.

26. NHS England. GP five year forward view. London: Department of Health; 2016.

27. King's College London: National Nursing Research Unit. Does NHS staff wellbeing affect patient experience of care? Policy plus evidence, issues and opinions in healthcare. 2013;May 2013(39).

28. Rimmer A. A third of GP training posts are vacant after first recruitment round London: BMJ; 2015 [Available from: http://careers.bmj.com/careers/advice/A_third_of_GP_training_posts_are_vacant_after_first_recruitment_round.

29. Rt Hon J Hunt MP Secretary of State for Health. Up to 1,500 extra medical training places announced London: UK Department of Health; 2016 [Available from: https://www.gov.uk/government/news/up-to-1500-extra-medical-training-places-announced.

For peer review only

**Figure 1 Career intentions to quit direct patient care by gender and age**

For peer review only

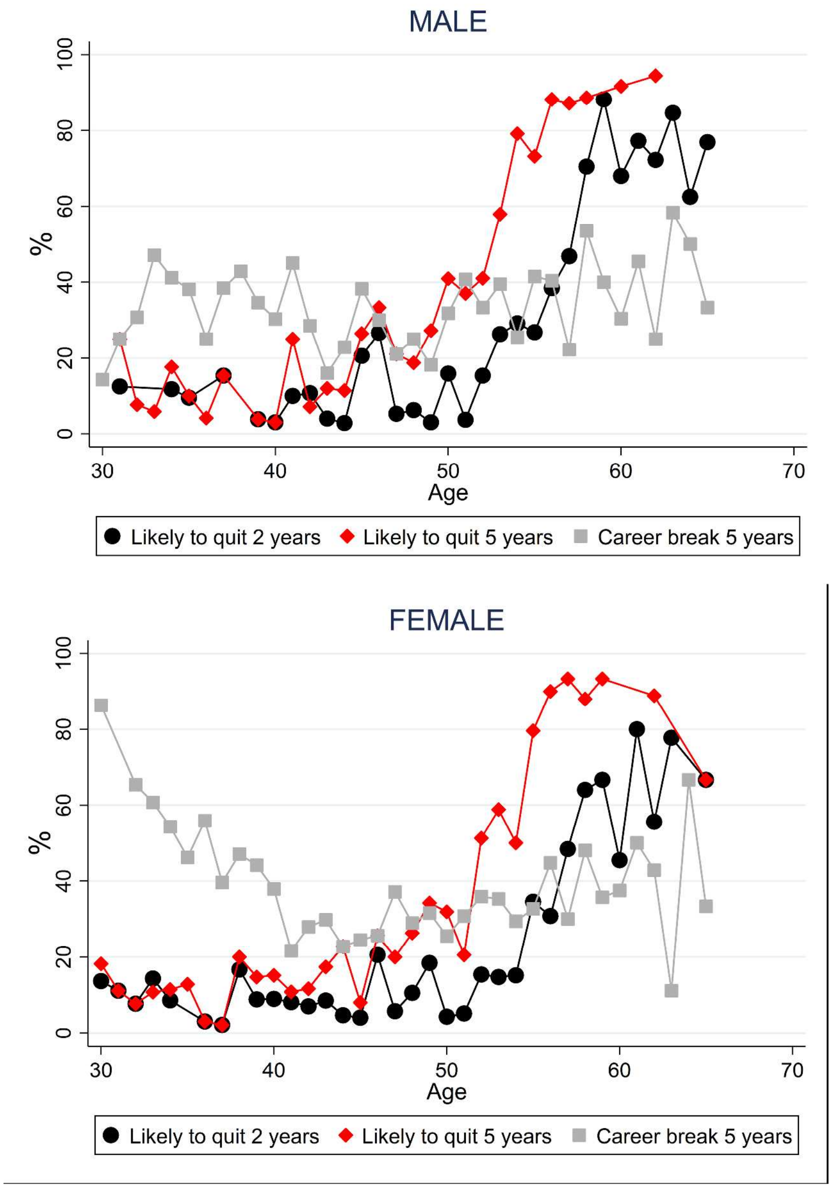


Figure 1

291x423mm (300 x 300 DPI)

**Supplementary Online Material Table 1. Sample characteristics**

| Characteristic (N/3370) | Responded n (%) |
|---|---|
| **Gender (3370)** | |
| Male | 1051 (67) |
| Female | 1197 (66) |
| **Age (years; 3370)** | |
| Under 40 | 500 (54) |
| 40-49 | 738 (68) |
| 50-54 | 391 (72) |
| 55-59 | 409 (76) |
| 60-69 | 192 (74) |
| 70 and over | 18 (75) |
| **Employment Status (3370)** | |
| GP provider | 1335 (71) |
| GP salaried | 394 (57) |
| Non-principal/locum | 519 (64) |
| **Survey sent by email (3370)** | |
| Yes | 1304 (71) |
| No | 944 (62) |
| **Postal address used (3370)** | |
| Practice address | 1744 (67) |
| Home address | 504 (64) |

For peer review only

National Institute for Health Research | University of Exeter Medical School | University of Bristol | University of Exeter Business School | Centre for Workforce Intelligence

# SUPPORTING THE GP WORKFORCE - ReGROUP QUESTIONNAIRE

**This questionnaire is part of a research study examining the issue of the GP workforce. Please help by completing the questionnaire. Please answer the questions below by ticking one box for each question and printing text where required in block capitals. We will keep your answers completely confidential.**

**If you would prefer to complete the survey online, please go to** ex.ac.uk/GP_Workforce

**Reference** 1234567890

**'Direct patient care' includes time spent in all routine general practice activities (in the UK), including consultations, telephone, clinical and practice administration, prescribing and referral.**

## GP WORKFORCE QUESTIONS

Q.1 **Please tell us which of the following scenarios applies to you?**
☐I am currently working in direct patient care
☐I am currently on a career break (i.e. a break of 6 months or more for maternity/paternity leave or personal/professional development, including doing medical work abroad)
☐I have permanently left direct patient care within the last 5 years................................**Go to Q.9**
☐None of the above...........................**Go to Q.9**

Q.2 **How likely is it that you will permanently leave direct patient care within the next 2 years?**
☐Very Likely
☐Likely
☐Unlikely
☐Very unlikely

Q.3 **How likely is it that you will permanently leave direct patient care within the next 5 years?**
☐Very Likely
☐Likely
☐Unlikely
☐Very unlikely

Q.4 **Overall, have you reduced your weekly average hours spent in direct patient care within the last 5 years?**
☐No
☐Yes

Q.5 **How likely is it that you will reduce your weekly average hours spent in direct patient care within the next 5 years?**
☐Very Likely
☐Likely
☐Unlikely

Q.6 **How likely is it that you will take a career break (or another career break) within the next 5 years?**
☐Very Likely
☐Likely
☐Unlikely
☐Very unlikely

Q.7 **How would you describe your current level of morale?**
☐Very Low
☐Low
☐Neither low nor high
☐High
☐Very high

Q.8 **Have you taken any steps toward changing your work-life balance (e.g. sought pension advice, consulted LMC, BMA etc)?**
☐No, I am still considering my options
☐Yes, I am in the process of making this change
☐Yes, I have made this change

## ABOUT YOU

Q.9 **What is your gender?**
☐ Male
☐ Female
☐ Prefer not to say

Q.10 **What is your age? (to nearest year)**

Q.11 **What year did you qualify in medicine? (YYYY)**

<RESEARCH ID>

Please turn over ☞
For peer review only - http://bmjopen.bmj.com/site/about/guidelines.xhtml

☐Very unlikely

**Q.12** **What year did you qualify as a GP? (YYYY)**

**Q.13** **From what region did you obtain your primary medical qualification?**
☐UK/Ireland
☐Europe (non-UK/Ireland)
☐South Asia
☐Other

**Q.14** **What is your ethnic group?**
☐White
☐Mixed/multiple ethnic groups
☐Asian/Asian British
☐Black/African/Caribbean/Black British
☐Other ethnic group
↳ **Please write in:**

**Q.15** **In your current/most recent direct patient care role, what is/was your position?**
☐GP Partner
☐Salaried GP
☐Locum GP
☐Other
↳ **Please write in:**

**Q.16** **In your current/most recent direct patient role, how many sessions do/did you work in a typical week? (Please consider a session as a block of approximately four hours of clinical time)**
↳ **Please write in:**

**sessions**

**Q.17** **When do you work these sessions in a typical week? Please tick the appropriate boxes:**

| | MON | TUE | WED | THU | FRI | SAT | SUN |
|---|---|---|---|---|---|---|---|
| Morning | ☐ | ☐ | ☐ | ☐ | ☐ | ☐ | ☐ |
| Afternoon | ☐ | ☐ | ☐ | ☐ | ☐ | ☐ | ☐ |
| Evening | ☐ | ☐ | ☐ | ☐ | ☐ | ☐ | ☐ |

**Q.18** **Do you have a practice that you are primarily affiliated to in your most recent direct patient care role?**
☐No
☐Yes
↳ **If yes, please write in the practice name AND ODS code (e.g. L123456), if known:**

**Q.19** **Are you personally involved in the delivery of out of hours direct patient care?**
☐Yes
☐No

## CAN WE TALK TO YOU?

**Q.20** **Would you be willing to take part in a confidential interview (45mins) to discuss issues around work-life balance? (You would be reimbursed for time)**
☐Yes
☐No

**Q.21** **We plan on developing policies and strategies to help support the experienced GP workforce. Would you agree to be approached by this research team for other research studies that address this agenda?**
☐Yes
☐No

**Q.22** **If you would be willing to be interviewed and/or be contacted for other research studies addressing the workforce agenda, please provide:**

***Preferred method of contact***

**Your email address:** ☐

______________________________________

**Your telephone number:** ☐

______________________________________

**Q.23** **What is your preferred time of day to be contacted?**

☐Morning
☐Lunchtime
☐Afternoon
☐Evening

**On:**
☐Weekdays
☐Weekends

## PRIZE DRAW

**Q.24** **Please tick the box below if you would like to be entered into a prize draw to receive one of five Kindles**
☐Yes, please enter me into the prize draw

**THANK YOU FOR YOUR TIME**
**Please return this questionnaire in the reply paid envelope provided (no stamp is needed)**

For peer review only - http://bmjopen.bmj.com/site/about/guidelines.xhtml

STROBE Statement—checklist of items that should be included in reports of observational studies

| | Item No | Recommendation |
|---|---|---|
| **Title and abstract** | 1 | (*a*) Indicate the study's design with a commonly used term in the title or the abstract **[Within the title page 1 and design section of the abstract page 2 ]** |
| | | (*b*) Provide in the abstract an informative and balanced summary of what was done and what was found **[See results section of abstract page 2 ]** |
| **Introduction** | | |
| Background/rationale | 2 | Explain the scientific background and rationale for the investigation being reported **[page 3-4]** |
| Objectives | 3 | State specific objectives, including any prespecified hypotheses **[page 4]** |
| **Methods** | | |
| Study design | 4 | Present key elements of study design early in the paper [**page 4]** |
| Setting | 5 | Describe the setting, locations, and relevant dates, including periods of recruitment, exposure, follow-up, and data collection **[page 4]** |
| Participants | 6 | (*a*) *Cohort study*—Give the eligibility criteria, and the sources and methods of selection of participants. Describe methods of follow-up<br>*Case-control study*—Give the eligibility criteria, and the sources and methods of case ascertainment and control selection. Give the rationale for the choice of cases and controls<br>*Cross-sectional study*—Give the eligibility criteria, and the sources and methods of selection of participants **[page 4]** |
| | | (*b*) *Cohort study*—For matched studies, give matching criteria and number of exposed and unexposed<br>*Case-control study*—For matched studies, give matching criteria and the number of controls per case |
| Variables | 7 | Clearly define all outcomes, exposures, predictors, potential confounders, and effect modifiers. Give diagnostic criteria, if applicable **[page 4]** |
| Data sources/ measurement | 8* | For each variable of interest, give sources of data and details of methods of assessment (measurement). Describe comparability of assessment methods if there is more than one group **[page 4-5]** |
| Bias | 9 | Describe any efforts to address potential sources of bias **[page 5]** |
| Study size | 10 | Explain how the study size was arrived at **[n/a]** |
| Quantitative variables | 11 | Explain how quantitative variables were handled in the analyses. If applicable, describe which groupings were chosen and why **[page 5-6]** |
| Statistical methods | 12 | (*a*) Describe all statistical methods, including those used to control for confounding [**page 5-6]** |
| | | (*b*) Describe any methods used to examine subgroups and interactions **[page 6]** |
| | | (*c*) Explain how missing data were addressed **[page 5]** |
| | | (*d*) *Cohort study*—If applicable, explain how loss to follow-up was addressed<br>*Case-control study*—If applicable, explain how matching of cases and controls was addressed<br>*Cross-sectional study*—If applicable, describe analytical methods taking account of sampling strategy **[n/a]** |
| | | (*e*) Describe any sensitivity analyses **[n/a]** |

Continued on next page



For peer review only - http://bmjopen.bmj.com/site/about/guidelines.xhtml

| | | |
|---|---|---|
| **Results** | | |
| Participants | 13* | (a) Report numbers of individuals at each stage of study—eg numbers potentially eligible, examined for eligibility, confirmed eligible, included in the study, completing follow-up, and analysed **[page 6]** |
| | | (b) Give reasons for non-participation at each stage **[page 6]** |
| | | (c) Consider use of a flow diagram **[n/a]** |
| Descriptive data | 14* | (a) Give characteristics of study participants (eg demographic, clinical, social) and information on exposures and potential confounders **[page 6-7]** |
| | | (b) Indicate number of participants with missing data for each variable of interest **[page 9]** |
| | | (c) *Cohort study*—Summarise follow-up time (eg, average and total amount) **[n/a]** |
| Outcome data | 15* | *Cohort study*—Report numbers of outcome events or summary measures over time |
| | | *Case-control study*—Report numbers in each exposure category, or summary measures of exposure |
| | | *Cross-sectional study*—Report numbers of outcome events or summary measures **[page 9]** |
| Main results | 16 | (*a*) Give unadjusted estimates and, if applicable, confounder-adjusted estimates and their precision (eg, 95% confidence interval). Make clear which confounders were adjusted for and why they were included **[page 12-14]** |
| | | (*b*) Report category boundaries when continuous variables were categorized **[page 12-14]** |
| | | (*c*) If relevant, consider translating estimates of relative risk into absolute risk for a meaningful time period **[n/a]** |
| Other analyses | 17 | Report other analyses done—eg analyses of subgroups and interactions, and sensitivity analyses **[page 11]** |
| **Discussion** | | |
| Key results | 18 | Summarise key results with reference to study objectives **[page 15]** |
| Limitations | 19 | Discuss limitations of the study, taking into account sources of potential bias or imprecision. **[page 16]** Discuss both direction and magnitude of any potential bias **[page 16]** |
| Interpretation | 20 | Give a cautious overall interpretation of results considering objectives, limitations, multiplicity of analyses, results from similar studies, and other relevant evidence **[page 16-17]** |
| Generalisability | 21 | Discuss the generalisability (external validity) of the study results **[page 16]** |
| **Other information** | | |
| Funding | 22 | Give the source of funding and the role of the funders for the present study and, if applicable, for the original study on which the present article is based **[page 19]** |

*Give information separately for cases and controls in case-control studies and, if applicable, for exposed and unexposed groups in cohort and cross-sectional studies.

**Note:** An Explanation and Elaboration article discusses each checklist item and gives methodological background and published examples of transparent reporting. The STROBE checklist is best used in conjunction with this article (freely available on the Web sites of PLoS Medicine at http://www.plosmedicine.org/, Annals of Internal Medicine at http://www.annals.org/, and Epidemiology at http://www.epidem.com/). Information on the STROBE Initiative is available at www.strobe-statement.org.

For peer review only
